# Inheritance through the cytoplasm

**DOI:** 10.1038/s41437-022-00540-2

**Published:** 2022-05-07

**Authors:** M. Florencia Camus, Bridie Alexander-Lawrie, Joel Sharbrough, Gregory D. D. Hurst

**Affiliations:** 1 Department of Genetics, Evolution and Environment, University College London, London, UK; 2 Biology Department, New Mexico Institute of Mining and Technology, Socorro, NM USA; 3 Institute of Infection, Veterinary and Ecological Sciences, University of Liverpool, Liverpool, England

**Keywords:** Evolutionary genetics, Genetic variation

## Abstract

Most heritable information in eukaryotic cells is encoded in the nuclear genome, with inheritance patterns following classic Mendelian segregation. Genomes residing in the cytoplasm, however, prove to be a peculiar exception to this rule. Cytoplasmic genetic elements are generally maternally inherited, although there are several exceptions where these are paternally, biparentally or doubly-uniparentally inherited. In this review, we examine the diversity and peculiarities of cytoplasmically inherited genomes, and the broad evolutionary consequences that non-Mendelian inheritance brings. We first explore the origins of vertical transmission and uniparental inheritance, before detailing the vast diversity of cytoplasmic inheritance systems across Eukaryota. We then describe the evolution of genomic organisation across lineages, how this process has been shaped by interactions with the nuclear genome and population genetics dynamics. Finally, we discuss how both nuclear and cytoplasmic genomes have evolved to co-inhabit the same host cell via one of the longest symbiotic processes, and all the opportunities for intergenomic conflict that arise due to divergence in inheritance patterns. In sum, we cannot understand the evolution of eukaryotes without understanding hereditary symbiosis.

## Introduction

The vast majority of genes in eukaryotes are located within chromosomal structures in the nucleus of the cell. These transmit copies of themselves to the next generation via meiosis involving strict segregation. However, eukaryotic cells also harbour smaller genomes, which reside in the cytoplasm, including: mitochondrial DNA, chloroplast DNA, and symbiont genomes. Interestingly, cytoplasmic genetic elements have been shown to have very different inheritance patterns to classic Mendelian nuclear chromosomes. The first documented evidence for this came from Carl Correns research on the four o’clock plant *Mirabilis jalapa*, in which he detailed the non-Mendelian inheritance of leaf colour (Correns 1909). Inheritance, in this case, was strictly maternal: a seed derived from an ovule from a non-green stem gave rise to non-green progeny, irrespective of the source of pollen. By 1952, the evidence of various forms of cytoplasmically inherited elements (CIEs) had grown, leading Joshua Lederberg to synthesise the inheritance of cellular organelles and symbionts into one framework in his treatise “Cell genetics and hereditary symbiosis” (Lederberg 1952). Furthermore, evidence for diversity in inheritance patterns (paternal or biparental) of CIEs started accumulating for a wide range of taxa (Birky 2001).

Since then, studies have demonstrated that CIEs are diverse and important - in many cases, encoding key aspects of organismal function. Cytoplasmically inherited elements vary in their level of integration with the host - in the case of organelles, the proteome is jointly encoded in nuclear and organellar DNA, in addition to integration into cellular physiology. For obligate microbial symbionts, anatomical and physiological integration are evident but generally without trafficking of host proteins into the microbe; they are commonly present in particular tissues and have host organised vertical transmission. Other microbial symbionts are less integrated, present more diffusely in the host and invade the germ line to gain vertical transmission.

**Fig. 1:**
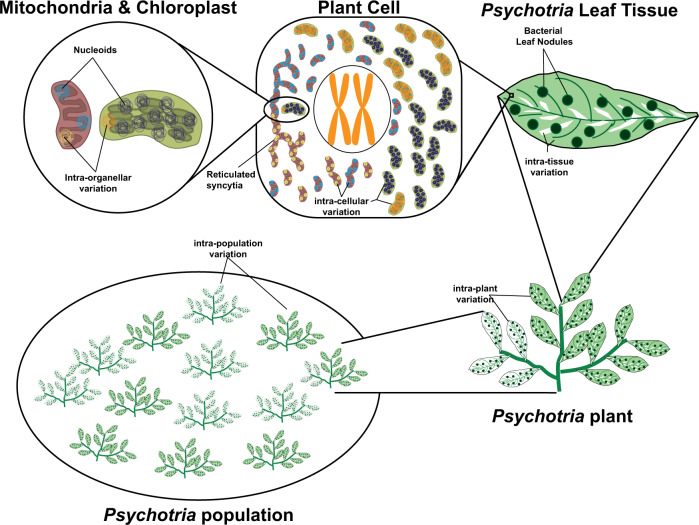
Cytoplasmically inherited elements produce variation at multiple scales of biological organisation. New mutations that arise immediately produce intra-organellar variation depicted here by differently coloured nucleoids (Mitochondria & Chloroplast panel). If mutations (differently coloured nucleoids) spread between organelles, variation between organelles is observed (Plant Cell panel). Note that mitochondria often form reticulated syncytia, rather than discrete compartments, in contrast to chloroplasts, which may facilitate recombination and therefore spread of mutations throughout the cell. Intracellular variation can give rise to intra-tissue variation, depicted here in the form of a variegated leaf (*Psychotria* leaf panel). *Psychotria* also features bacterial leaf nodules (dark green circles) that contain *Burkholderia* bacteria which are vertically inherited through the seed. Variation within tissues can then give rise to variation across tissues (*Psychotria* Plant panel). If germlines are segregated late, this can result in distinct alleles being propagated to the next generation from different parts of the plant. As a consequence, the variation that originated at the individual organelle level can finally be observed between individuals within populations (*Psychotria* population panel).

In this review, we describe the diversity of inheritance systems of CIEs, and highlight the evolutionary consequences that these inheritance systems bring to cellular, organismal and population dynamics (Fig. 1). For this, we focus on the three main groups of CIEs: mitochondrial DNA, chloroplast DNA, and symbiont genomes. We begin by outlining the origins of cytoplasmic inheritance and the evolution of uniparental inheritance, documenting the diversity of cytoplasmic inheritance systems so far observed. We discuss the diversity and patterns of genome organisation for cytoplasmic elements and examine the population genetics of CIEs, highlighting the tension between within- and between-individual spread. We summarise the evidence for the adaptive importance of cytoplasmic genes before detailing coadaptation between the cytoplasm and the nucleus, and amongst cytoplasmic components.

## The evolutionary origins of vertical transmission and uniparental inheritance

The ancestors of current cytoplasmically inherited genetic material were free-living organisms (Sagan 1967), but how cytoplasmic inheritance originated and came to be limited to one sex remains an open question. For microeukaryotes (and indeed for the ancestral protoeukaryote) the presence of a microbe inside the cytoplasm would *de facto* produce inheritance on cell division. This would form a continuous system if replication of the microbe within the cell was occurring. Thus, the primary drive to cytoplasmic inheritance is intracellular location and replication, which could be initially host driven (symbiont capture) or symbiont driven (infection of the host, or escape from a phagolysosome).

Notably, the rate of vertical transmission for symbionts that can also transmit infectiously is evolvable: symbionts with mixed modes of transmission that are kept in continuously growing host populations (where cell division provides ample opportunity for vertical transmission) evolve a stronger tendency for heritable transmission compared to those kept in populations at carrying capacity (where the opportunity for vertical transmission is limited) (Magalon et al. 2010). Thus, there is a trajectory in which infection through the environment is lost.

When vertical transmission does evolve, there are two primary consequences. First, the population size of symbionts and mixing of strains declines, reducing within host conflicts. Second, symbiont fitness becomes a product not only of host survival but additionally host reproduction. Both of these processes drive the symbiosis towards the mutualism end of the mutualism-parasitism continuum, with current models indicating restrictions on symbiont diversity through bottlenecks and reduced mixing opportunities being most important in this transition, through quelling the conflicts associated with within-host competition (Leeks et al. 2019). Later transitions would then involve adaptation to the intracellular environment with correlated loss of capacity for free-living life and infection processes. All of these are reflected in the reductive genome evolution pattern commonly observed in heritable symbionts (Moran et al. 2008).

The evolution of inheritance for symbionts of multicellular hosts also has its origins in the association of free-living organisms, with a transition from symbiosis where the parties reform symbiosis through environmental association each generation to vertical transmission. Indeed, some symbiont clades include both symbionts acquired through the environment and heritable symbionts (e.g., (Drew et al. 2021)). Vertical transmission may arise passively through spatial structure (symbionts from a parent are more likely to infect progeny of that parent through proximity), actively through selection on the host to ensure passage of a beneficial symbiont (host driven vertical transmission) or actively through selection on the microbe to infect the next generation through the germ line. Evolution towards vertical transmission may be constrained through location (e.g., soil microbe-plant root associations have no proximity to the germ line), and made less likely in environments where partner availability is high (e.g., aquatic environments (Douglas 1998)) or where the symbiont has utility only in a restricted part of host life history (Hartmann et al. 2017).

Most cytoplasmic genetic material is inherited uniparentally - that is to say from one parent only. It is notable that uniparental inheritance is not restricted to anisogamous species - it is also commonly observed in isogamous microeukaryotes associated with mating types. This observation implies that uniparental inheritance is not a simple by-product of gamete size, but rather is an evolved state (Hurst and Hamilton 1992). Anisogamy itself is considered by some as a potential onward adaptive mechanism for imposing uniparental inheritance, in contrast to the passive view that uniparental inheritance is a by-product of anisogamy (Hurst 1990).

Evolutionary drivers of uniparental inheritance include the benefit of preventing conflicts and the damage from cytoplasmic mixing. Under a biparental inheritance scenario, we would expect a heteroplasmic state, in which multiple distinct forms of the CIE exist within the same cell, to be the norm. For mitochondria, heteroplasmy interferes with cell functioning, with empirical work demonstrating that it can cause organismal dysfunction (Nissanka and Moraes 2020). Moreover, heteroplasmy reduces the variance between cells, and if this is happening in the germ line, it then reduces the efficacy of selection (Radzvilavicius et al. 2016). Mathematical models show that selection against heteroplasmy can lead to the fixation of uniparental inheritance in an ancestrally biparental population (Christie et al. 2015; Christie and Beekman 2017). Mathematical models indicate that mitonuclear coadaptation is improved with uniparental inheritance and mitochondrial bottlenecks under a wide range of conditions (Hadjivasiliou et al. 2012).

## Beyond simple maternal inheritance

Whilst the majority of anisogamous species transmit CIEs uniparentally, via the egg (Birky 2001), heteroplasmy via paternal leakage can occur when maternal inheritance is not strictly enforced (see Table 1 and Supplementary Table  S1 for examples). Most species appear to exhibit some degree of leakage if sampled carefully enough (e.g., (Wagner et al. 1991; Kvist et al. 2003; Fontaine et al. 2007; Bentley et al. 2010; Nunes et al. 2013)), but the processes that contribute to variation in leakage rates are not well understood. By contrast, a variety of mechanisms that reinforce maternal inheritance by eliminating and/or silencing paternally derived elements have been documented (Sato and Sato 2013, 2017). Still, paternally derived CIEs occasionally experience positive selection, resulting in introgression and even replacement of the maternal CIE lineage by the paternal CIE lineage (Wolff et al. 2013).

**Table 1:** Alternative cytoplasmic inheritance mechanisms observed in plant, fungal, and animal mitochondria and chloroplasts.

Inheritance pattern	Cytoplasmic element	Representative taxa	Reference
Paternal leakage	Mitochondria	Mouse	(Gyllensten et al. 1991)
Paternal leakage	Chloroplasts	*Brassicaceae*	(Schneider et al. 2015)
Paternal inheritance	Mitochondria	*Cucumis sativus (cucumber)*	(Havey 1997)
Paternal inheritance	Chloroplasts	*Pinus taeda* (loblolly pine)	(Neale and Sederoff 1989)
Maternal leakage	Chloroplasts	*Pinus radiata* (Monterey pine)	(Cato and Richardson 1996)
Divergent heteroplasmy	Mitochondria	*Sphenodon punctatus* (tuatara)	(Macey et al. 2021)
Biparental inheritance	Mitochondria	*Saccharomyces cerevisiae* (Baker’s yeast)	(Birky et al. 1978)
Biparental inheritance	Chloroplasts	*Oenothera*	(Chiu et al. 1988)
Doubly-uniparental inheritance	Mitochondria	*Mytilus edulis* (blue mussel)	(Skibinski et al. 1994)

See Supplementary Table S1 for expanded view.

Notably, there exists a biological distinction between paternal leakage versus paternal inheritance of CIEs. Paternal leakage, in which CIEs are inherited mostly maternally, but with some minor contribution from the paternal gamete, provides at least some opportunity for genetic exchange between CIEs from different lineages. By contrast, CIE lineages from separate parents are not expected to interact or recombine under systems of paternal inheritance, even if only occasional, in which all of the CIEs present in an individual host are paternally derived (e.g., Ross et al. 2016). The implications are that paternal leakage allows for breakdown of linkage disequilibrium between separate nucleotides of the CIE genome, such that beneficial mutations can be decoupled from deleterious genomic backgrounds and vice versa (Hill and Robertson 1966).

Evolutionary transitions from maternal to paternal inheritance are not especially common, but do happen (Table 1, Supplementary Table S1). Interestingly, shifts in one organelle do not always affect the other cytoplasmic elements within the cell (but see *Pelargonium* (Weihe et al. 2009); *Sequoia* (Neale et al. 1989)), indicating that the genetic machinery regulating cytoplasmic inheritance is independent across separate CIEs. For example, in *Musa acuminata* (banana), the mitochondria are inherited paternally and the chloroplasts are inherited maternally (Fauré et al. 1994). Some other examples of mixed uniparental inheritance include cucumbers, melons (Havey 1997) (in contrast to the rest of the *Cucurbitaceae* (Havey et al. 1998)), which follow the same pattern as banana, and loblolly pines, which feature maternally transmitted mitochondria and paternally transmitted chloroplasts (Neale and Sederoff 1989).

When both parents contribute CIEs to the offspring, it is probable that cells benefit from biparental mitochondrial inheritance as it provides higher individual genetic diversity, which leads to reduced susceptibility to deleterious mutation. However, this naturally leads to competition and conflict between lineages. Recent work has shown that biparental inheritance has the potential to be beneficial and remain stable for hybridising populations if the fitness cost of mitonuclear incompatibilities in hybrids is greater than a stable state of heteroplasmy (which is also detrimental to the individual, but less so) (Allison et al. 2021). In hybridised *Pelargonium* (geranium) cultivars, for example, mitochondrial and plastid biparental inheritance has been seen alongside chloroplast variegation (Baur 1908). In these hybrids, differing ratios of maternally and paternally derived cytoplasmic genomes were observed across different tissues (Weihe et al. 2009). Based on the phylogenetic distribution of biparental inheritance systems, it seems that the balance of reduced impact of deleterious mutations vs. increased conflict between CIE lineages favours the latter, as relatively few examples of fully biparentally inherited CIEs exist.

A particularly interesting case of biparental inheritance of mitochondria is that of doubly-uniparental inheritance (DUI) in bivalves, in which the paternal mtDNA (M-type) is passed down to male offspring and maternal mtDNA (F-type) is passed down to offspring of both sexes (Breton et al. 2007; Passamonti and Ghiselli 2009; Zouros 2013). In this scheme, two independently evolving mtDNA lineages are found in male individuals, but the sperm produced only contain the lineage they inherited paternally (Ladoukakis and Zouros 2017). The paternal mitochondria are largely confined to the gonad and the maternal mitochondria the soma, resulting in sperm that carry only the male mitochondrial line (Ghiselli et al. 2020). The separate localisation of M-type vs. F-type mtDNAs also makes it less likely that recombination between mtDNA lineages occurs (but see (Zouros 2000; Burzyński et al. 2003; Passamonti et al. 2003; Breton et al. 2006; Stewart et al. 2009)).

Heritable microbes are commonly considered as exclusively transmitted from mother to offspring only; however, this rule is not absolute. Many heritable viruses show biparental inheritance, commonly with higher fidelity through the egg than sperm (Roossinck 2010). For heritable bacteria, early studies of *Volvox carteri* revealed efficient paternal inheritance of what are now known to be *Cand*. Megaira symbionts (Lee and Kochert 1976); later work showed biparental inheritance of *Rickettsia* in *Nephotettix* planthoppers (Watanabe et al. 2014), and *Sodalis glossinidia* in tsetse fly hosts (De Vooght et al. 2015). Biparental inheritance allows symbionts to drive to high frequency without either conferring a benefit to their host, or exhibiting reproductive parasitism. It also creates an environment where mixed infections become common, which may lead to the evolution of increased virulence (associated with competition for transmission) and also potentiates recombination.

## Genomic organisation and interactions with the nuclear genome

### Genomic architectural organisation and variation in CIEs

Cytoplasmically inherited genomes are highly variable across eukaryotes in terms of both size and structure (Smith and Keeling 2015). Mitochondrial and chloroplast genomes are rarely lost entirely (but see (Hjort et al. 2010; Keeling 2010)); however, the diminutive size of mtDNAs in *Plasmodium* species (~6 kb) (Hikosaka et al. 2011) compared to the massive and multi-partite mitochondrial genomes of *Silene conica* (~11.3 Mb) (Sloan et al. 2012) and *Larix siberica* (~11.7 Mb) (Putintseva et al. 2020) highlight the diverse trajectories of cytoplasmic genome evolution following the original endosymbiotic event and subsequent massive transfer to the nucleus prior to the Last Eukaryotic Common Ancestor (Sloan et al. 2018).

Plastid genomes are less variable in size than mitochondrial genomes (Wu et al. 2020), but non-photosynthetic plastids have seen dramatic reductions in size compared to their photosynthetic ancestors (de Koning and Keeling 2006; Barbrook et al. 2014). While many CIEs exhibit circular genomes (e.g., most bilaterian mitochondrial genomes (Boore 1999) and most eubacterial symbionts), linear (Stampar et al. 2019; Escalante et al. 1998; Nosek et al. 2004; Shao et al. 2009), branched (Oldenburg and Bendich 1996), and multi-chromosomal arrangements (Wu et al. 2020) have arisen multiple separate times across eukaryotes. The absence of Mendelian inheritance in cytoplasmic genomes likely contributes to the tremendous variation observed there, to the extent that once multi-chromosomal genomes evolve, their inheritance is highly fragmented and inconsistent (Wu and Sloan 2019).

In parallel, heritable symbiont genomes vary greatly in genome complexity. There is a general pattern of genome reduction in symbiotic microbes, first associated with the transition from environmentally acquired to heritable, and then with the transition from facultative (not required by the host) to obligate (required by the host). Obligately required heritable symbionts have genomes that are commonly <1 Mb, and may be as small as 112 kb (Bennett and Moran 2013), in comparison to free-living microbes with genome sizes >4 Mb. Pseudogenization is rapid during the first phases of evolution, often accompanied by proliferation of mobile elements (Bennett and Moran 2013). G/C content typically reduces as the genome shrinks, with obligate symbiont genomes typically highly AT rich (Moran et al. 2008).

### Interactions and molecular cross talk with the nuclear genome

It is common to observe genes in the nuclear genome which have cytoplasmic origin, and transfer of material from both mitochondria and microbial symbionts to the nucleus is ongoing (Bensasson et al. 2001; Dunning Hotopp et al. 2007). Importantly, recent nuclear derived symbiont sequences on occasion have strong phenotypic effects, potentiating retention (Leclercq et al. 2016). In deep evolutionary time, these transfers fuelled the seemingly inevitable gene transfer from the cytoplasm to the nucleus and subsequent genome streamlining that CIEs have repeatedly undergone (Timmis et al. 2004; Giannakis et al. 2021). This process has resulted in the vast majority of proteins that function in cytoplasmically inherited organelle compartments being encoded by the nucleus (Millar 2007; Meisinger et al. 2008; van Wijk and Baginsky 2011; Muthye and Lavrov 2018). The genes and gene products that are still retained in CIE genomes must therefore physically interact with nuclear-encoded gene products. To wit, four of the five multi-subunit enzymes that comprise the electron transport chain and the photosynthetic enzyme complexes of chloroplasts feature intimate interactions between subunits encoded by separately inherited and expressed genomes (Rand et al. 2004; Forsythe et al. 2019).

Much attention has been paid to the molecular nature of these cytoplasmic-nuclear interactions (Osada and Akashi 2011; van der Sluis et al. 2015; Beck et al. 2015; Adrion et al. 2016; Mossman et al. 2017; Rand and Mossman 2020; Evans et al. 2021), but relatively little is known about the stoichiometry of these interactions, except that cytoplasmic gene expression is consistently higher than expression of nuclear-encoded genes involved in the same multi-subunit complexes (Havird and Sloan 2016). Further, mitochondrial DNA depletion is associated with a number of different diseases in humans (Blokhin et al. 2008; Clay Montier et al. 2009; Monickaraj et al. 2012; Petersen et al. 2014; Pyle et al. 2016; Tin et al. 2016; Ashar et al. 2017; Liu et al. 2020), and polyploid plants exhibit elevated cytoplasmic DNA content per cell compared to diploid relatives to maintain cytonuclear stoichiometry following genome doubling (Fernandes Gyorfy et al. 2021). Even from this currently limited understanding, it is clear that complex stoichiometric relationships exist between the nuclear and cytoplasmic genomes and gene products, and perturbations to cytonuclear stoichiometry can therefore have drastic consequences for the cells that experience them.

## Population and evolutionary genetics of cytoplasmic elements

### Mutations and how they spread throughout the cytoplasm

Cytoplasmically inherited elements present a stark contrast to Mendelian traits as sources of variation. As Bill Birky noted, CIEs are ‘non-stringent’ genetic traits that can vary in *quantity* as well as sequence – in contrast to Mendelian elements limited to a copy number of two in any diploid cell (Birky 2001). Instead, CIEs are often highly multi-copy within cells (Kukat et al. 2011; Carelli et al. 2015; Schaack et al. 2020). As such, the distribution of sequence variation in CIEs is profoundly affected by the distribution of copy number variation, as mutants must first establish within the pool inside a cell, then amongst the cells within an individual, then amongst individuals in the population. Variation in copy number within the cell is also critical to the stoichiometric balance between the cytoplasmic and nuclear genomes, as they contribute to the assembly of the multi-subunit enzyme complexes that carry out bioenergetic processes like photosynthesis and respiration (Forsythe et al. 2019).

Mutational spread in cytoplasmically inherited genomes is fundamentally dependent upon the rate of occurrence of new mutations (Sung et al. 2012; Waneka et al. 2021). However, the multi-copy nature of CIEs makes it practically impossible to determine their absolute mutation rates (Schaack et al. 2020). Nevertheless, a large effort across decades has been dedicated to quantifying relative mitochondrial mutation rates and frequency spectra (Brown et al. 1979; Wolfe et al. 1987; Denver et al. 2000; Haag-Liautard et al. 2008; Howe et al. 2009; Havird and Sloan 2016; Allio et al. 2017; Konrad et al. 2017; Wu et al. 2020; Broz et al. 2021; Waneka et al. 2021), providing valuable information about the extent of heteroplasmy caused by de novo mutations (Waneka et al. 2021) and the probability of transmitting those heteroplasmies to the next generation (Konrad et al. 2017). Mutational spectra of mtDNA are also important to mutational spread (reviewed in (Katju and Bergthorsson 2019)). For example, oxidation of guanines (i.e., 8-oxo-G), especially in mitochondria, can result in elevated CG → AT transversions through mispairing with adenine (Cheng et al. 1992; Kino et al. 2017).

Despite the aforementioned difficulty in ascertaining absolute mutation rates in CIEs, it is clear that CIE mutation rates vary tremendously across taxa. For example, animal mtDNAs exhibit substantially higher mutation rates than plant mtDNAs (Wolfe et al. 1987). Indeed, animal mtDNA mutation rate varies more than two orders of magnitude across taxa (Nabholz et al. 2007). Certain plant lineages have shown episodic accelerations in cytoplasmic genome mutation rates (Sloan et al. 2014; Sloan 2015; Havird et al. 2015; Williams et al. 2019; Broz et al. 2021). There is also tremendous variation in mutation rate across different cellular genomes – animal mtDNAs exhibit higher mutation rates than animal nuclear genomes (Brown et al. 1979; Wolfe et al. 1987; Havird and Sloan 2016), but plant nuclear genomes exhibit higher mutation rates than plant cpDNA and plant mtDNAs (Wolfe et al. 1987).

Whilst mutation rate is not known for heritable microbes, it is known they vary substantially in substitution rate, with some heritable microbes evolving at a rate comparable to viruses (Gerth et al. 2021), and others, like *Wolbachia*, two-three orders of magnitude slower (Richardson et al. 2012). At least some of this variation can be traced back to the different mechanisms of replication and repair across taxa and compartments (Brown et al. 2005; Maréchal and Brisson 2010; Lewis et al. 2015; Gerth et al. 2021), all of which have implications for mutation rate (Longley et al. 2005; DeBalsi et al. 2017; Wu et al. 2020).

### Mutational masking in cytoplasmic elements

New mutations that arise in CIEs face a dramatically different population genetic landscape compared to Mendelian elements because there are typically many competing cytoplasmic genomes present inside each cell, and because organellar cytoplasmic elements do not experience segregation, as is the case for nuclear genomes during sexual reproduction (Wilton et al. 2018). Thus, new mutations face a steep drift barrier, with their effects on host function being masked until reaching higher frequency within a cell or individual (potentially as high as 80% (King and Attardi 1989; Boulet et al. 1992; Stewart and Chinnery 2015)). As a consequence, mutations that exist at low frequencies among CIEs in a parent are likely to be lost as a result of the bottleneck that occurs between generations in multicellular organisms.

There are two sides to the mutational masking that results from harbouring many copies of cytoplasmic genomes inside cells: (1) mutations with deleterious fitness effects on the host can persist longer than they otherwise would if maintained in single-copy form within the cell (Otto 2007), and (2) mutations with beneficial fitness effects on the host can be lost at higher rates because their effects are largely invisible to selection. Recent high-resolution efforts support the existence of mutational masking, as nonsynonymous mutations are more common and exist at higher frequencies than expected (Waneka et al. 2021). Moreover, masking of the fitness effects of mutations is expected to result in a deletion bias (Lawless et al. 2020), especially under relaxed selection (Wickett et al. 2008), as CIEs with replication advantages (e.g., CIEs with smaller genomes) can rise in frequency within cells rapidly (Wallace 1989; Clark et al. 2012; Sloan and Wu 2014). This latter pattern may contribute to the observation that CIEs exhibit more streamlined genomes compared to their free-living relatives (Timmis et al. 2004; Giannakis et al. 2021).

The population of CIEs within cells, of cells within tissues, of somatic vs. germ line tissues, and of individual hosts within host populations gives rise to the expectation of multi-level selection, in which elements that have an advantage in terms of spread at a lower level of organisation do not necessarily possess the same advantage at higher levels of organisation (Fig. 1). To wit, mutations that remain at low frequencies across generations in human mtDNA can rise to high frequency in separate tissues within the same individual (Samuels et al. 2013; Rebolledo-Jaramillo et al. 2014; Li et al. 2015). Additionally, recent work in which artificial mixed infections of *Buchnera* were created within aphids demonstrated a ‘regular winner’, despite strong drift effects - but the winner did not necessarily confer individual level benefits (Perreau et al. 2021).

### Recombination in the cytoplasm

The misconception that CIEs do not undergo recombination has been largely debunked. For example, phylogenetic and other experimental analyses of animal mitochondrial genomes consistently recover signatures of inter-molecular recombination, indicating that inheritance leakage may play a major role in CIE genome evolution (Mita et al. 1990; Kajander et al. 2000; Ladoukakis and Zouros 2001; Ladoukakis and Eyre-Walker 2004; Piganeau et al. 2004; Barr et al. 2005; Ciborowski et al. 2007; Ma and O’Farrell 2015; Leducq et al. 2017; Dahal et al. 2018). Plant plastids and plant mitochondria exhibit recombination-directed repair (Cerutti et al. 1995; Day and Madesis 2007; Maréchal and Brisson 2010; Davila et al. 2011; Gualberto and Newton 2017; Chevigny et al. 2020; Wu et al. 2020), as well as rampant structural rearrangement via repeat-mediated recombination (Palmer 1983; Ogihara et al. 1988; Palmer and Herbon 1988; Gray et al. 1999; Arrieta-Montiel and Mackenzie 2011; Cole et al. 2018; Wu and Sloan 2019; Xia et al. 2020). This latter phenomenon, termed substoichiometric shifting, makes for extensive, but heritable structural variation within individuals (Woloszynska 2009; Maréchal and Brisson 2010; Arrieta-Montiel and Mackenzie 2011; Davila et al. 2011).

Recombination has played such a large role in plant CIEs that their relatively slow rate of molecular evolution is thought to be due, at least in part, to recombination (Palmer and Herbon 1988; Chevigny et al. 2020; Wu et al. 2020), as heteroplasmies may be eliminated from intracellular populations via gene conversion. Whether the occasional and episodic accelerations in rates of cytoplasmic genome evolution in some plant lineages (Williams et al. 2019; Broz et al. 2021) is associated with altered recombinatorial activity remains an open question. Heritable microbes also show clear signatures of recombination (Baldo et al. 2006), as well as acquisition of genetic material from other bacteria (Nikoh et al. 2014), which commonly involves phage transfer (Kaur et al. 2021; Boyd et al. 2021).

The evolutionary consequences of cytoplasmic recombination are profound: recombination can act as a barrier to new mutations through gene conversion and can facilitate the rise of beneficial mutations and the elimination of deleterious mutations by separating those mutations from their genomic backgrounds (Neiman and Taylor 2009), particularly when distinct CIEs occur inside the same cell or organism.

### Cytoplasmic adaptation

The seemingly asexual nature of animal cytoplasmic genomes suggests that they, like other asexual elements, will experience impaired adaptive evolution. Experimental evidence, however, has found this not to be the case, as numerous studies have reported signatures of positive selection acting within the mitochondrial genome (Mishmar et al. 2003; Ruiz-Pesini et al. 2004; Meiklejohn et al. 2007). Furthermore, clinal patterns in mtDNA genomes have been detected across several species, indicating signatures of adaptation (Camus et al. 2017; Silva et al. 2014). Early studies by Lynch and Blanchard (1998) found that mitochondrial genes had higher ratios of nonsynonymous to synonymous mutations in relation to the nuclear genome of plants, invertebrate and fungal taxa (Lynch and Blanchard 1998). Most recently, Morales et al. (2015) found evidence for positive selection on several amino acids in the mtDNA of the Australian eastern yellow robin (*Eopsaltria australis*) populations. The authors additionally found nuclear genome homogeneity within the robin populations sampled indicating that there were high levels of gene flow, thus the signatures of positive selection were unique to the mtDNA (Morales et al. 2015). The combined outcomes of these studies suggest that certain mtDNA protein-coding genes of natural populations might well have been shaped by positive selection.

Less evidence is available linking chloroplast genomes to adaptive processes, but this could be because of the slower rates of evolution, higher levels of complexity or the fact that dissecting the contributions of multiple organelle genomes is complicated. Nevertheless, there has been some work in domesticated species testing these questions. For example, research on rice (*Oryza*) has identified 14 chloroplast genes with strong signatures of positive selection, with these genes being mainly related to photosynthetic function. Interestingly, authors found that eight of these genes were independently found in sun-loving species, whereas other photosynthetic genes were selected in shade-tolerating species (Gao et al. 2019). Other studies have directly examined the effects of cytonuclear interactions across two different ecological environments. The sunflower genus *Helianthus* is commonly used as a model as many of its species have adapted to very distinct niches (Levin 2003). Sambatti and colleagues performed reciprocal transplant experiments between *H. annus* and *H. petiolaris* which inhabit mesic and xeric habitats respectively (Sambatti et al. 2008). In addition to examining both coevolved strains, authors used all possible backcross combinations to dissect the contribution of the cytoplasm and nuclear genome, finding that the cytoplasm was the main driver for fitness, and is therefore adapted to these two contrasting environments (Sambatti et al. 2008).

Heritable symbionts have been commonly observed to be under strong selection. Invasion of heritable symbionts into populations in real time has been observed on numerous occasions. For instance, a classic example is the wave of *Wolbachia* that induced cytoplasmic incompatibility (CI) which swept through Californian *D. simulans* populations in the 1980s (Turelli and Hoffmann 1991). Similarly, heritable *Spiroplasma* that provide tolerance to nematode parasitism have spread through North America, and *Rickettsia* spreading through whitefly populations has been observed over the last 20 years (Himler et al. 2011; Shi et al. 2021). It is also common to observe that symbionts either themselves have low diversity - or that associated mitochondria have low diversity - implying a recent history of joint selection. Finally, it is notable that heritable microbes (unlike mitochondria and chloroplasts) may segregate during host reproduction, with a fraction of progeny not inheriting them. Their maintenance thus requires some form of drive - either a benefit to host survival or reproductive parasitism. As such, it is argued they are never neutral traits, but are maintained by a selection - segregational loss balance (Jaenike 2012). In addition, they present different modularities of adaptive variation - like other CIEs they have different circulating variants in a population, but in addition, these commonly exist alongside uninfected cytotypes, which are the equivalent of a null allele.

## Coadaptation

### Coadaptation with nuclear encoded proteins and systems

The transfer of genetic material from cytoplasmic elements to the nucleus is thought to have created strong pressures for both nuclear and cytoplasmic genomes to cooperate with one another. Excessive amounts of conflict can have severe consequences to both host and symbiont. One of the classic demonstrations of mitonuclear coadaptation comes from studies using hybrid crosses from natural populations. The copepod species *Tigriopus californicus* has become one of the main wild model systems, primarily due to the high level of intraspecific divergence in mtDNA genomes. While crosses between populations give F1 offspring with normal (if not elevated) fitness compared to the parental generation, there is a drastic decrease in fitness in the F2 generations and beyond (Burton 1990). Using backcrossing approaches, they discovered that this decrease in fitness was caused by severe mitonuclear incompatibilities (Burton and Lee 1994), and the proportion of the maternal nuclear genome appears to be positively correlated with developmental rate in backcrossed individuals (Han and Barreto 2021). The nuclear-encoded mitochondrial genes (those interacting with genes encoded in the mtDNA) of the *T. californicus* genome have also been shown to coadapt with mtDNA, exhibiting elevated mutation-rate-corrected rates of evolution (i.e., *d*_*N*_*/d*_*S*_) compared to the rest of the nuclear-encoded DNA, matching the rapid pace of evolution in their mtDNA counterparts (Barreto et al. 2018).

Similarly, chloroplast genomes are predicted to be under strong selection to coadapt, and much work has been done to document the effects of plastid-nuclear interactions on plant fitness (Greiner et al. 2011; Postel and Touzet 2020). Nearly all of the ~75–80 proteins encoded by the chloroplast genome are involved in protein complexes which exhibit important functions that are essential to plant function, such as Rubisco and photosystems I and II. One of the best examples of coadaptation between chloroplast and nuclear genomes after intraspecific hybridisation comes from the genus *Oenothera* (Stubbe 1989; Greiner and Bock 2013), in which three basic haploid nuclear genomes can be paired with five different chloroplast genomes; giving a total of 30 possible chloro-nuclear combinations. Of these, only 12 produce a viable green phenotype, whereas the 18 remaining associations lead to various degrees of cytonuclear incompatibilities, from reduced phenotypic capacity to embryo lethality (Cleland 1972; Dietrich et al. 1997). Subsequent work suggests that the radiation within *Oenothera* is approximately 1 million years old, suggesting that incompatibilities and coadaptation mechanisms have rapidly evolved (Greiner et al. 2008). Although most chloroplast genomes evolve relatively slowly, occasional accelerations in evolutionary rate have occurred throughout angiosperms (Williams et al. 2019). In these cases, the nuclear-encoded interacting partners of chloroplast-encoded proteins exhibit corresponding increases in evolutionary rate, reflecting the co-evolutionary dynamics of plastid-nuclear interactions (Bock et al. 2014; Zhang et al. 2015; Dai et al. 2016; Weng et al. 2016; Rockenbach et al. 2016; Havird et al. 2017; Li et al. 2019; Forsythe et al. 2021).

Heritable symbionts are distinct from organelles in their interaction with the host in that symbiont proteomes are generally considered to be encoded within their genomes, rather than jointly with the nuclear genome. Thus, this route to coadaptation is less important. Nevertheless, symbionts have profound interplay with their host in terms of cell biology, organismal development, physiology and anatomy. In particular, many heritable symbionts form obligate partnerships with their host - such that neither party can live alone. For beneficial symbionts, there are systems to control symbiont number through antimicrobial production (Login et al. 2011), development of specific systems for housing and transmitting symbionts, alongside membrane systems/transporters for metabolite exchange (Feng et al. 2019), which all represent adaptations on the host to house and maintain symbiosis. On the symbiont side, there is loss of cell walls and pathways required for growth outside of the host environment, leading to reliance on host supply of nutrients. An interesting phenomenon is dependence - where the host cannot live without the symbiont for reasons other than the services supplied by the symbiont. One of the first cases of hereditary microbial symbiosis - between bacteria and plants of the family *Rubiaceae* - is one of these, where loss of the symbiont was observed to impede host development (Miller 1990). Further cases include the requirement of *Asobara tabida* for a particular strain of *Wolbachia* to complete oogenesis (Dedeine et al. 2001). These cases likely represent the host evolving around the presence/products of the symbiont, such that symbiont removal results in loss of function.

### Coadaptation across the cytoplasm

The study of cytoplasmically inherited agents has acknowledged the diversity of genetic material in the cytoplasm, but rarely examined interactions between the parties. Interactions may be either direct (e.g., a mito-symbiont interaction), or indirect (an evolutionary response in one that impacts the other through coinheritance). Whilst little is known about the former, evidence of indirect impacts is plentiful - selection on one party feeds through to the other inherited elements. For example, the spread of *Wolbachia* causing CI through a population carries the linked mtDNA haplotype, and the selective sweep reduces mtDNA diversity at the population level (Turelli et al. 1992; Hurst and Jiggins 2005; Deng et al. 2021). Indeed, there are a variety of cases where symbionts are thought to have driven the movement of mtDNA across species boundaries (Turelli et al. 1992; Hurst and Jiggins 2005; Deng et al. 2021). Following spread, the presence of a symbiont at equilibrium in the population has been considered to reduce the effective population of mtDNA to that associated with the fraction which carries the symbiont, conceptually equivalent to background selection removing certain individuals from the pool of individuals out of which mutations arise and spread. More recently, it has been argued the reciprocal pattern is also likely - selective sweeps on mtDNA impact diversity (and indeed frequency and presence) of symbionts (Fenton et al. 2021). Thus, the diversity and population genetics of the cytoplasm should be taken summatively, rather than simply with regard to individual elements.

## Conflicts between cytoplasmically inherited elements and their hosts

Maternal inheritance produces an association between symbiont fitness and that of their female, but not male host. This has two primary consequences - the mother’s curse (selection optimises CIEs to female phenotype fitness) and reproductive parasitism (selection optimises CIEs to maximise the production and survival of infected daughters).

### Mother’s curse

**Fig. 2:**
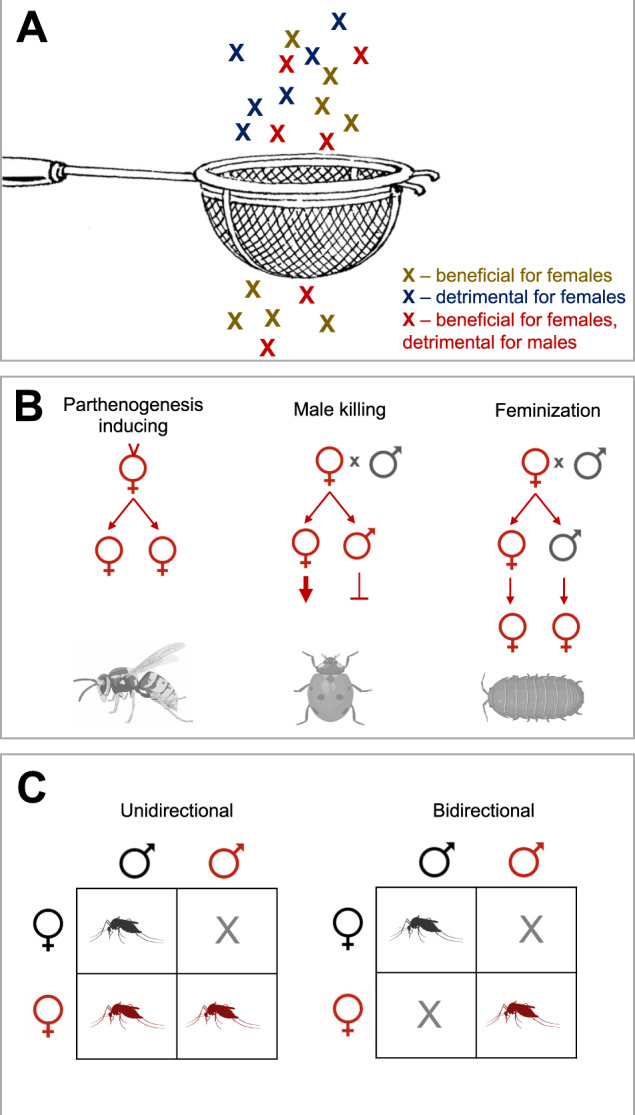
Conflicts between cytoplasmically inherited elements and their hosts. Differences in inheritance patterns between nuclear and cytoplasmic elements provides an arena for intergenomic conflict. **A** Mothers curse hypothesis: maternal inheritance of mitochondria can result in the accumulation of mutations with sexually antagonistic effects in the mtDNA genome. **B** Cytoplasmic sex ratio distortion in species with separate sexes. Commonly, investment into male and female offspring is equal. Maternal inheritance ties symbiont fitness to the production and survival of female hosts. This is manifested in parthenogenesis induction (left), where all progeny are daughters. Male-killing (middle), where the symbiont kills male progeny it enters, and sibling females have greater access to resources, and higher survival, as a result. Feminisation (right), where the symbiont impacts development in progeny that have a male karyotype such that they differentiate as female hosts. **C** Cytoplasmic incompatibility is the result of severe miscommunication between cytoplasmic and nuclear genomes, and a classic example of how cytoplasmic elements can spread through a population. When hosts carrying the symbiont (red) mate with uninfected hosts (grey), CI can result in inviable offspring in a unidrectional (left) or bidirectional (right) fashion.

The theoretical framework for the mother’s curse hypothesis was first described in the 1990s (Frank and Hurst 1996), with further theoretical support proposed a decade later. This framework is simple; the uniparental maternal inheritance of mtDNA means that males are prone to inherit mutations that are selected through the female lineage, even if these mutations are detrimental to males. Consequently, males are expected to accumulate these sexually antagonistic mutations over evolutionary time (Fig. 2A).

The first experimental evidence for mother’s curse came from a *Drosophila* study that examined the effects of mtDNA genetic variation on the transcriptomic response (Innocenti et al. 2011). This study used cybrids (cytoplasmic hybrids), in which the mitochondrial genomes from five fly strains sourced from different parts of the world were coupled independently to an isogenic nuclear background, decoupling the effects of mtDNA from those of the nuclear genome. Approximately 10% of the nuclear genome was differentially expressed in males, whereas only a handful of genes were affected in females. Interestingly, these differentially expressed genes were particularly involved in the male reproductive system (testes, accessory glands, ejaculatory duct). Another clear example of mother’s curse came from humans in which a male-biased mutation in the mtDNA resulted in Leber’s hereditary optical neuropathy (Milot et al. 2017). The particular mutation was tracked over a 290-year period, by identifying via genealogical records that it was first recorded in Canada from a woman arriving from France in the 1600s. Given the large male fitness consequences conferred by the mutation, natural selection would be expected to remove this variant from the population, but authors noticed a slight increase in frequency, suggesting a female fitness benefit (Milot et al. 2017). More recently though, the scope of mother’s curse has broadened to not only include reproductive traits, but also other sex-specific life-history traits (Montooth and Dhawanjewar 2019; Nagarajan-Radha et al. 2020; Carnegie et al. 2021).

What is not currently clear is the extent to which mother’s curse impacts maternally inherited elements beyond mitochondria. Conceptually, beneficial heritable symbioses are expected to experience a parallel process in terms of adaptation - the capacity of a maternally inherited symbiont to protect a male host, for instance, derives solely from correlated selection from its impact on female hosts. However, the degree to which maternally inherited beneficial symbioses perform less well in male hosts has not been investigated.

### Reproductive parasitism: Investment into female over male progeny and gametes

Both symbionts and mitochondria are known to bias the pattern of host investment into, and survival of, female hosts/gametes over male (see (Hurst and Frost 2015) for review). In hermaphroditic plants, mitochondrial variants impact the development of anthers and pollen formation in the phenotype of cytoplasmic male sterility (CMS). These variants divert resources to reproduction through ovule/seed, and in so doing, promote their own transmission (Fig. 2B).

In arthropods, symbionts variously induce parthenogenetic reproduction (thus ensuring all progeny are female and can transmit the element), feminise hosts that are otherwise ‘programmed’ to male development, or selectively kill male hosts they enter. This last phenotype appears to be spiteful, but is actually an adaptive phenotype when the death of male hosts releases resources directly (through consumption) or indirectly (through relaxed competition) to sibling females (Hurst and Majerus 1993). In ladybirds, for instance, dead male eggs (through which the symbiont cannot be transmitted) are consumed by their sisters (which carry the symbiont and can transmit them). Embryonic male-killing in dioecious species is thus conceptually equivalent to CMS in hermaphrodites, as a source of resource reallocation from male to female reproduction. Male-killing may also occur later in development, and here it is commonly associated with infectious transmission of symbionts from male hosts, with the symbiont showing mixed modes of transmission (maternal inheritance through females, infectious transmission through males (Hurst 1991).

The sex ratio/allocation distorting phenotypes described above have impacts on the individual, but also strong ecological and evolutionary consequences. Parthenogenesis inducing symbionts can spread to fixation, converting the species from sexual to asexual (Stouthamer et al. 1990). Cytoplasmic male sterility, feminisation and male-killing agents can cause strongly female-biased population sex ratios, and these may alter patterns of sexual selection (Jiggins et al. 2000; Charlat et al. 2007) and indeed the capacity of a host to effectively reproduce (Dyson and Hurst 2004). Perhaps most importantly, they engender strong selection on their hosts to restore sex allocation/sex ratio to parity. This is reflected in nuclear restorer genes against mitochondria inducing CMS (Frank 1989), and suppressor genes rescuing male function against male-killers (Hornett et al. 2006) or preventing transmission of feminizers (Rigaud and Juchault 1992). The strongly female-biased sex ratios created when cytoplasmic sex-ratio distorters are common creates intense Fisherian selection for suppression/restorer elements, such that the spread of suppression/restorer genes represents some of the strongest selective events recorded in natural populations (e.g., (Charlat et al. 2007; Hornett et al. 2014)). Further, the evolution of suppression may hide the underlying reproductive parasitism, which may only become apparent in crosses between populations (Hornett et al. 2006), hybridisation (Frank 1989), or for symbionts, transinfection to a novel host species (Sasaki et al. 2002). Indeed, the commonness with which CMS suppression evolves is reflected in the emergence of CMS in about 20% of hybridisation events in plants (Frank 1989).

There are several open questions in our understanding of sex ratio distortion. In terms of incidence, mitochondrial sex allocation distorters are commonly observed in plants, but not in animals. This may be associated with differences in coding capacity/genetics of plant vs. animal genomes (mutational constraint). Symbionts that distort sex ratio/allocation are very commonly observed in arthropods, and have been hypothesised as present in sea urchins (Carrier et al. 2021). Given heritable microbes are common, it is expected that sex ratio distortion would be present in a wider array of host than is currently recognised. In terms of impact, a key emerging question is the nature of restorer and suppression mutations. What are the host systems that are impacted in this co-evolutionary arms race? It has been widely hypothesised these may involve modifications of the sex determination system, as alterations of the signal or target of the symbiont (Hornett et al. 2014). This awaits further discovery of the mechanism of male-killing and of suppression; however, that symbionts alter splicing of key sex determination genes like *doublesex* supports sex determination as a focus for suppression (Sugimoto et al. 2010).

### Reproductive parasitism: Cytoplasmic incompatibility

Cytoplasmic Incompatibility (CI) phenotypes describe the failure of zygote development where the male parent is infected with a symbiont and the female parent either does not have that symbiont, or carries a different strain of the symbiont. Originally described as a phenotype of the *Wolbachia* in arthropods (Yen and Barr 1971), this phenotype has since been associated with diverse insect symbionts, including *Cardinium* and *Rickettsiella* (Hunter et al. 2003; Rosenwald et al. 2020). The symbionts conferring CI spread as it imposes a cost solely on uninfected lineages; the positive frequency-dependent nature of the advantage means invasion either requires the symbiont to reach a threshold frequency, or has an alternate phenotype that allows initial establishment. In contrast to symbionts with sex ratio distorting phenotypes, the symbiont becomes less costly to the host when it is common, simply because symbiont-infected females are unaffected. Indeed, hosts are selected to retain the symbiont to provide immunity against CI when the symbiont is common (Fig. 2C).

Cytoplasmic Incompatibility is a very important phenotype for two reasons. First, it can be applied in the form of release of infected males to suppress target vector populations, and in the form of inoculative release of strains that combine CI and suppress viral replication, to reduce vectorial capacity. Thus, basic research on cytoplasmic symbionts (Hedges et al. 2008; Teixeira et al. 2008) has translated into applied public health protection measures (Utarini et al. 2021). Second, spread of a CI inducing symbiont in one population or species may provide a unidirectional barrier to hybridisation against another, and the spread of distinct strains may produce a bidirectional barrier (Bordenstein et al. 2001). In both cases, the symbiont spread induces reproductive isolation and thus potentiates speciation.

## Conclusions

In 1919, Thomas Hunt Morgan in his Principles of Heredity wrote:

‘That there may be substances in the cytoplasm that propagate themselves and are outside the influence of the nucleus, must, of course, be at once conceded as possible despite the fact that, aside from certain plastids, all the Mendelian evidence fails to show that there are such characters’. (Morgan 1919)

By contrast, the past 100 years have created awareness of the vast amount of genetic biodiversity found inhabiting the cytoplasm. Very early on it was noted that these genetic elements did not follow Mendelian inheritance patterns, with inheritance being mostly maternal. One topic that remains a long-standing and unresolved question are why most organelle genomes transmit maternally? Since the initial observations of CIEs, many exceptions to this rule have been discovered, from paternal inheritance of mtDNA in cucumbers, to doubly-uniparental inheritance in bivalves to biparental inheritance in yeast, and symbionts combining infectious and vertical transmission. Still, a predominance for cytoplasmic elements to be inherited via the maternal lineage is evident from the phylogenetic record. The tight association of cytoplasmic genomes with the rest of the cell, plus the non-Mendelian inheritance patterns of CIEs results in fascinating co-evolutionary dynamics that manifest at multiple scales of biological organisation. Thus, the diversity, inheritance, and functional roles of CIEs across eukaryotes remain an important and open question in biology, with fundamental implications for the cells and organisms in which they reside.

## Supplementary information

Supplementary Information
