# Supplementary material for: Inheritance through the cytoplasm
Source: Heredity (Edinb). 2022 May 7;129(1):31–43. doi: 10.1038/s41437-022-00540-2 (PMC9273588; doi:10.1038/s41437-022-00540-2)
Supplement: Supplementary file 1 — Supplementary Information [file 41437_2022_540_MOESM1_ESM.pdf]

Table S1. List of taxa that display evidence of non-maternal inheritance of mitochondria or chloroplast DNA.

Inheritance Pattern	Cytoplasmic Element	Taxonomic Group	Species Name (common name)	Reference
Biparental Inheritance	Mitochondria	Animals	*Carassius auratus x Cyprinus carpio allotriploid*	Guo, X., Liu, S., & Liu, Y. (2006). Evidence for recombination of mitochondrial DNA in triploid crucian carp. *Genetics*, *172*(3), 1745-1749.
Biparental Inheritance	Mitochondria	Animals	*Engraulis encrasicolus (anchovy)*	Magoulas, A., & Zouros, E. (1993). Restriction-site heteroplasmy in anchovy (Engraulis encrasicolus) indicates incidental biparental inheritance of mitochondrial DNA.
Biparental Inheritance	Mitochondria	Animals	*Schistosoma mansoni*	Jannotti-Passos, L. K., Souza, C. P., Parra, J. C., & Simpson, A. J. G. (2001). Biparental mitochondrial DNA inheritance in the parasitic trematode Schistosoma mansoni. *Journal of Parasitology*, *87*(1), 79-82.
DUI	Mitochondria	Animals	*Actinonaias ligamentina*	Hoeh, W. R., Stewart, D. T., & Guttman, S. I. (2002). High fidelity of mitochondrial genome transmission under the doubly uniparental mode of inheritance in freshwater mussels (Bivalvia: Unionoidea). *Evolution*, *56*(11), 2252-2261.
DUI	Mitochondria	Animals	*Amblema plicata*	Curole, J. P., & Kocher, T. D. (2005). Evolution of a unique mitotype-specific protein-coding extension of the cytochrome c oxidase II gene in freshwater mussels (Bivalvia: Unionoida). *Journal of Molecular Evolution*, *61*(3), 381-389.
DUI	Mitochondria	Animals	*Anodonta anatina*	Soroka, M. (2010). Characteristics of mitochondrial DNA of unionid bivalves (Mollusca: Bivalvia: Unionidae). I. Detection and characteristics of doubly uniparental inheritance (DUI) of unionid mitochondrial DNA. *Folia malacologica*, *18*(4).
DUI	Mitochondria	Animals	*Anodonta californensis*	Mock, K. E., Brim-Box, J. C., Miller, M. P., Downing, M. E., & Hoeh, W. R. (2004). Genetic diversity and divergence among freshwater mussel (Anodonta) populations in the Bonneville Basin of Utah. *Molecular Ecology*, *13*(5), 1085-1098.
DUI	Mitochondria	Animals	*Anodonta implicata*	Curole, J. P., & Kocher, T. D. (2002). Ancient sex-specific extension of the cytochrome c oxidase II gene in bivalves and the fidelity of doubly-uniparental inheritance. *Molecular Biology and Evolution*, *19*(8), 1323-1328.
DUI	Mitochondria	Animals	*Anodonta oregonensis*	Mock, K. E., Brim-Box, J. C., Miller, M. P., Downing, M. E., & Hoeh, W. R. (2004). Genetic diversity and divergence among freshwater mussel (Anodonta) populations in the Bonneville Basin of Utah. *Molecular Ecology*, *13*(5), 1085-1098.
DUI	Mitochondria	Animals	*Anodonta wahlamatensis*	Mock, K. E., Brim-Box, J. C., Miller, M. P., Downing, M. E., & Hoeh, W. R. (2004). Genetic diversity and divergence among freshwater mussel (Anodonta) populations in the Bonneville Basin of Utah. *Molecular Ecology*, *13*(5), 1085-1098.
DUI	Mitochondria	Animals	*Anodonta woodiana*	Soroka, M. (2008). Doubly uniparental inheritance of mitochondrial DNA in the freshwater bivalve Anodonta woodiana (Bivalvia: Unionidae). *Folia Biologica (Krakow)*, *56*(1-2), 91-95.
DUI	Mitochondria	Animals	*Arctica islandica*	Dégletagne, C., Abele, D., & Held, C. (2016). A distinct mitochondrial genome with DUI-like inheritance in the ocean quahog Arctica islandica. *Molecular biology and evolution*, *33*(2), 375-383.
DUI	Mitochondria	Animals	*Brachidontes exustus*	Lee, T., & Ó FOIGHIL, D. (2004). Hidden Floridian biodiversity: mitochondrial and nuclear gene trees reveal four cryptic species within the scorched mussel, Brachidontes exustus, species complex. *Molecular Ecology*, *13*(11), 3527-3542.

DUI	Mitochondria	Animals	*Brachidontes pharaonis*	Lee, T., & Ó FOIGHIL, D. (2004). Hidden Floridian biodiversity: mitochondrial and nuclear gene trees reveal four cryptic species within the scorched mussel, Brachidontes exustus, species complex. *Molecular Ecology* , *13* (11), 3527-3542.
DUI	Mitochondria	Animals	*Brachidontes variabilis*	Terranova, M. S., Lo Brutto, S., Arculeo, M., & Mitton, J. B. (2007). A mitochondrial phylogeography of Brachidontes variabilis (Bivalvia: Mytilidae) reveals three cryptic species. *Journal of Zoological Systematics and Evolutionary Research* , *45* (4), 289-298.
DUI	Mitochondria	Animals	*Cumberlandia monodonta*	Breton, S., Stewart, D. T., Shepardson, S., Trdan, R. J., Bogan, A. E., Chapman, E. G., ... & Hoeh, W. R. (2011). Novel protein genes in animal mtDNA: a new sex determination system in freshwater mussels (Bivalvia: Unionoida)?. *Molecular Biology and Evolution* , *28* (5), 1645-1659.
DUI	Mitochondria	Animals	*Cyclina sinensis*	*AB040833, AB040834*
DUI	Mitochondria	Animals	*Cyprogenia alberti*	Walker, J. M., Curole, J. P., Wade, D. E., Chapman, E. G., Bogan, A. E., Watters, G. T., & Hoeh, W. R. (2006). Taxonomic distribution and phylogenetic utility of gender-associated mitochondrial genomes in the Unionoida (Bivalvia). *MALACOLOGIA-PHILADELPHIA-* , *48* (1/2), 265.
DUI	Mitochondria	Animals	*Cyrtonaias tampicoensis*	Hoeh, W. R., Stewart, D. T., & Guttman, S. I. (2002). High fidelity of mitochondrial genome transmission under the doubly uniparental mode of inheritance in freshwater mussels (Bivalvia: Unionoidea). *Evolution* , *56* (11), 2252-2261.
DUI	Mitochondria	Animals	*Dahurinaia dahurica*	Walker, J. M., Curole, J. P., Wade, D. E., Chapman, E. G., Bogan, A. E., Watters, G. T., & Hoeh, W. R. (2006). Taxonomic distribution and phylogenetic utility of gender-associated mitochondrial genomes in the Unionoida (Bivalvia). *MALACOLOGIA-PHILADELPHIA-* , *48* (1/2), 265.
DUI	Mitochondria	Animals	*Donax cuneatus*	*AB040841, AB040842*
DUI	Mitochondria	Animals	*Donax faba*	*AB040843, AB040844*
DUI	Mitochondria	Animals	*Donax trunculus*	Theologidis, I., Fodelianakis, S., Gaspar, M. B., & Zouros, E. (2008). Doubly uniparental inheritance (DUI) of mitochondrial DNA in Donax trunculus (Bivalvia: Donacidae) and the problem of its sporadic detection in Bivalvia. *Evolution: International Journal of Organic Evolution* , *62* (4), 959-970.
DUI	Mitochondria	Animals	*Dromus dromas*	Walker, J. M., Curole, J. P., Wade, D. E., Chapman, E. G., Bogan, A. E., Watters, G. T., & Hoeh, W. R. (2006). Taxonomic distribution and phylogenetic utility of gender-associated mitochondrial genomes in the Unionoida (Bivalvia). *MALACOLOGIA-PHILADELPHIA-* , *48* (1/2), 265.
DUI	Mitochondria	Animals	*Ellipsaria lineolata*	Walker, J. M., Curole, J. P., Wade, D. E., Chapman, E. G., Bogan, A. E., Watters, G. T., & Hoeh, W. R. (2006). Taxonomic distribution and phylogenetic utility of gender-associated mitochondrial genomes in the Unionoida (Bivalvia). *MALACOLOGIA-PHILADELPHIA-* , *48* (1/2), 265.
DUI	Mitochondria	Animals	*Elliptio dilitata*	Walker, J. M., Curole, J. P., Wade, D. E., Chapman, E. G., Bogan, A. E., Watters, G. T., & Hoeh, W. R. (2006). Taxonomic distribution and phylogenetic utility of gender-associated mitochondrial genomes in the Unionoida (Bivalvia). *MALACOLOGIA-PHILADELPHIA-* , *48* (1/2), 265.

DUI	Mitochondria	Animals	*Epioblasma brevidens*	Walker, J. M., Curole, J. P., Wade, D. E., Chapman, E. G., Bogan, A. E., Watters, G. T., & Hoeh, W. R. (2006). Taxonomic distribution and phylogenetic utility of gender-associated mitochondrial genomes in the Unionoida (Bivalvia). *MALACOLOGIA-PHILADELPHIA-*, *48*(1/2), 265.
DUI	Mitochondria	Animals	*Fusconaia flava*	Hoeh, W. R., Stewart, D. T., Sutherland, B. W., & Zouros, E. (1996). Multiple origins of gender-associated mitochondrial DNA lineages in bivalves (Mollusca: Bivalvia). *Evolution*, *50*(6), 2276-2286.
DUI	Mitochondria	Animals	*Geukensia demissa*	Hoeh, W. R., Stewart, D. T., Sutherland, B. W., & Zouros, E. (1996). Multiple origins of gender-associated mitochondrial DNA lineages in bivalves (Mollusca: Bivalvia). *Evolution*, *50*(6), 2276-2286.
DUI	Mitochondria	Animals	*Glebula rotundata*	Curole, J. P., & Kocher, T. D. (2005). Evolution of a unique mitotype-specific protein-coding extension of the cytochrome c oxidase II gene in freshwater mussels (Bivalvia: Unionoida). *Journal of Molecular Evolution*, *61*(3), 381-389.
DUI	Mitochondria	Animals	*Gonidea angulata*	Walker, J. M., Curole, J. P., Wade, D. E., Chapman, E. G., Bogan, A. E., Watters, G. T., & Hoeh, W. R. (2006). Taxonomic distribution and phylogenetic utility of gender-associated mitochondrial genomes in the Unionoida (Bivalvia). *MALACOLOGIA-PHILADELPHIA-*, *48*(1/2), 265.
DUI	Mitochondria	Animals	*Hamiota subangulata*	Chapman, E. G., Piontkivska, H., Walker, J. M., Stewart, D. T., Curole, J. P., & Hoeh, W. R. (2008). Extreme primary and secondary protein structure variability in the chimeric male-transmitted cytochrome c oxidase subunit II protein in freshwater mussels: Evidence for an elevated amino acid substitution rate in the face of domain-specific purifying selection. *BMC Evolutionary Biology*, *8*(1), 1-16.
DUI	Mitochondria	Animals	*Hyridella menziesi*	Hoeh, W. R., Stewart, D. T., & Guttman, S. I. (2002). High fidelity of mitochondrial genome transmission under the doubly uniparental mode of inheritance in freshwater mussels (Bivalvia: Unionoidea). *Evolution*, *56*(11), 2252-2261.
DUI	Mitochondria	Animals	*Hyriopsis cumingii*	KC471519
DUI	Mitochondria	Animals	*Hyriopsis schlegelii*	Fukata, Y., & Iigo, M. (2020). F-type complete mitochondrial genomes of two Hyriposis species, H. schlegelii and H. cumingii (Bivalvia; Unionoida; Unionidae) from Lake Kasumigaura, Japan, and its phylogenetic analysis. *Mitochondrial DNA Part B*, *5*(2), 1218-1219.
DUI	Mitochondria	Animals	*Inversidens japanensis*	Doucet-Beaupré, H., Breton, S., Chapman, E. G., Blier, P. U., Bogan, A. E., Stewart, D. T., & Hoeh, W. R. (2010). Mitochondrial phylogenomics of the Bivalvia (Mollusca): searching for the origin and mitogenomic correlates of doubly uniparental inheritance of mtDNA. *BMC Evolutionary Biology*, *10*(1), 1-19.
DUI	Mitochondria	Animals	*Lamprotula leai*	Zieritz, A., Froufe, E., Bolotov, I., Gonçalves, D. V., Aldridge, D. C., Bogan, A. E., ... & Lopes-Lima, M. (2021). Mitogenomic phylogeny and fossil-calibrated mutation rates for all F-and M-type mtDNA genes of the largest freshwater mussel family, the Unionidae (Bivalvia). *Zoological Journal of the Linnean Society*, *193*(3), 1088-1107.
DUI	Mitochondria	Animals	*Lamprotula tortuosa*	Wang, G., Cao, X., & Li, J. (2013). Complete F-type mitochondrial genome of Chinese freshwater mussel Lamprotula tortuosa. *Mitochondrial DNA*, *24*(5), 513-515.

DUI	Mitochondria	Animals	*Lampsilis cardium*	Walker, J. M., Curole, J. P., Wade, D. E., Chapman, E. G., Bogan, A. E., Watters, G. T., & Hoeh, W. R. (2006). Taxonomic distribution and phylogenetic utility of gender-associated mitochondrial genomes in the Unionoida (Bivalvia). *MALACOLOGIA-PHILADELPHIA-*, *48*(1/2), 265.
DUI	Mitochondria	Animals	*Lampsilis hydiana*	Walker, J. M., Curole, J. P., Wade, D. E., Chapman, E. G., Bogan, A. E., Watters, G. T., & Hoeh, W. R. (2006). Taxonomic distribution and phylogenetic utility of gender-associated mitochondrial genomes in the Unionoida (Bivalvia). *MALACOLOGIA-PHILADELPHIA-*, *48*(1/2), 265.
DUI	Mitochondria	Animals	*Lampsilis ovata*	Chapman, E. G., Piontkivska, H., Walker, J. M., Stewart, D. T., Curole, J. P., & Hoeh, W. R. (2008). Extreme primary and secondary protein structure variability in the chimeric male-transmitted cytochrome c oxidase subunit II protein in freshwater mussels: Evidence for an elevated amino acid substitution rate in the face of domain-specific purifying selection. *BMC Evolutionary Biology*, *8*(1), 1-16.
DUI	Mitochondria	Animals	*Lampsilis powellii*	Walker, J. M., Curole, J. P., Wade, D. E., Chapman, E. G., Bogan, A. E., Watters, G. T., & Hoeh, W. R. (2006). Taxonomic distribution and phylogenetic utility of gender-associated mitochondrial genomes in the Unionoida (Bivalvia). *MALACOLOGIA-PHILADELPHIA-*, *48*(1/2), 265.
DUI	Mitochondria	Animals	*Lampsilis reeveiana*	Walker, J. M., Curole, J. P., Wade, D. E., Chapman, E. G., Bogan, A. E., Watters, G. T., & Hoeh, W. R. (2006). Taxonomic distribution and phylogenetic utility of gender-associated mitochondrial genomes in the Unionoida (Bivalvia). *MALACOLOGIA-PHILADELPHIA-*, *48*(1/2), 265.
DUI	Mitochondria	Animals	*Lampsilis siliquoidea*	Walker, J. M., Curole, J. P., Wade, D. E., Chapman, E. G., Bogan, A. E., Watters, G. T., & Hoeh, W. R. (2006). Taxonomic distribution and phylogenetic utility of gender-associated mitochondrial genomes in the Unionoida (Bivalvia). *MALACOLOGIA-PHILADELPHIA-*, *48*(1/2), 265.
DUI	Mitochondria	Animals	*Lampsilis straminea*	Curole, J. P., & Kocher, T. D. (2002). Ancient sex-specific extension of the cytochrome c oxidase II gene in bivalves and the fidelity of doubly-uniparental inheritance. *Molecular Biology and Evolution*, *19*(8), 1323-1328.
DUI	Mitochondria	Animals	*Lampsilis streckeri*	Walker, J. M., Curole, J. P., Wade, D. E., Chapman, E. G., Bogan, A. E., Watters, G. T., & Hoeh, W. R. (2006). Taxonomic distribution and phylogenetic utility of gender-associated mitochondrial genomes in the Unionoida (Bivalvia). *MALACOLOGIA-PHILADELPHIA-*, *48*(1/2), 265.
DUI	Mitochondria	Animals	*Lampsilis teres*	Hoeh, W. R., Stewart, D. T., & Guttman, S. I. (2002). High fidelity of mitochondrial genome transmission under the doubly uniparental mode of inheritance in freshwater mussels (Bivalvia: Unionoidea). *Evolution*, *56*(11), 2252-2261.
DUI	Mitochondria	Animals	*Lanceolaria grayana*	AB040829, AB040830
DUI	Mitochondria	Animals	*Lasmigona complanata*	Stewart, D. T., Hoeh, W. R., Bauer, G., & Breton, S. (2013). Mitochondrial genes, sex determination and hermaphroditism in freshwater mussels (Bivalvia: Unionoida). In *Evolutionary biology: exobiology and evolutionary mechanisms* (pp. 245-255). Springer, Berlin, Heidelberg.

DUI	Mitochondria	Animals	*Lasmigona costata*	Stewart, D. T., Hoeh, W. R., Bauer, G., & Breton, S. (2013). Mitochondrial genes, sex determination and hermaphroditism in freshwater mussels (Bivalvia: Unionoida). In *Evolutionary biology: exobiology and evolutionary mechanisms* (pp. 245-255). Springer, Berlin, Heidelberg.
DUI	Mitochondria	Animals	*Ledella sublevis*	Boyle, E. E., & Etter, R. J. (2013). Heteroplasmy in a deep-sea protobranch bivalve suggests an ancient origin of doubly uniparental inheritance of mitochondria in Bivalvia. *Marine Biology* , *160* (2), 413-422.
DUI	Mitochondria	Animals	*Ledella ultima*	Boyle, E. E., & Etter, R. J. (2013). Heteroplasmy in a deep-sea protobranch bivalve suggests an ancient origin of doubly uniparental inheritance of mitochondria in Bivalvia. *Marine Biology* , *160* (2), 413-422.
DUI	Mitochondria	Animals	*Lemiiox rimosus*	Chapman, E. G., Piontkivska, H., Walker, J. M., Stewart, D. T., Curole, J. P., & Hoeh, W. R. (2008). Extreme primary and secondary protein structure variability in the chimeric male-transmitted cytochrome c oxidase subunit II protein in freshwater mussels: Evidence for an elevated amino acid substitution rate in the face of domain-specific purifying selection. *BMC Evolutionary Biology* , *8* (1), 1-16.
DUI	Mitochondria	Animals	*Leptodea fragilis*	Walker, J. M., Curole, J. P., Wade, D. E., Chapman, E. G., Bogan, A. E., Watters, G. T., & Hoeh, W. R. (2006). Taxonomic distribution and phylogenetic utility of gender-associated mitochondrial genomes in the Unionoida (Bivalvia). *MALACOLOGIA-PHILADELPHIA-* , *48* (1/2), 265.
DUI	Mitochondria	Animals	*Leptodea leptodon*	Walker, J. M., Curole, J. P., Wade, D. E., Chapman, E. G., Bogan, A. E., Watters, G. T., & Hoeh, W. R. (2006). Taxonomic distribution and phylogenetic utility of gender-associated mitochondrial genomes in the Unionoida (Bivalvia). *MALACOLOGIA-PHILADELPHIA-* , *48* (1/2), 265.
DUI	Mitochondria	Animals	*Ligumia recta*	Hoeh, W. R., Stewart, D. T., & Guttman, S. I. (2002). High fidelity of mitochondrial genome transmission under the doubly uniparental mode of inheritance in freshwater mussels (Bivalvia: Unionoidea). *Evolution* , *56* (11), 2252-2261.
DUI	Mitochondria	Animals	*Limecola balthica*	Capt, C., Bouvet, K., Guerra, D., Robicheau, B. M., Stewart, D. T., Pante, E., & Breton, S. (2020). Unorthodox features in two venerid bivalves with doubly uniparental inheritance of mitochondria. *Scientific reports* , *10* (1), 1-13.
DUI	Mitochondria	Animals	*Margaritifera hembeli*	Curole, J. P., & Kocher, T. D. (2005). Evolution of a unique mitotype-specific protein-coding extension of the cytochrome c oxidase II gene in freshwater mussels (Bivalvia: Unionoida). *Journal of Molecular Evolution* , *61* (3), 381-389.
DUI	Mitochondria	Animals	*Margaritifera margaritifera*	Hoeh, W. R., Stewart, D. T., & Guttman, S. I. (2002). High fidelity of mitochondrial genome transmission under the doubly uniparental mode of inheritance in freshwater mussels (Bivalvia: Unionoidea). *Evolution* , *56* (11), 2252-2261.
DUI	Mitochondria	Animals	*Margaritifera marrianae*	Stewart, D. T., Hoeh, W. R., Bauer, G., & Breton, S. (2013). Mitochondrial genes, sex determination and hermaphroditism in freshwater mussels (Bivalvia: Unionoida). In *Evolutionary biology: exobiology and evolutionary mechanisms* (pp. 245-255). Springer, Berlin, Heidelberg.
DUI	Mitochondria	Animals	*Medionidus conradicus*	Walker, J. M., Curole, J. P., Wade, D. E., Chapman, E. G., Bogan, A. E., Watters, G. T., & Hoeh, W. R. (2006). Taxonomic distribution and phylogenetic utility of gender-associated mitochondrial genomes in the Unionoida (Bivalvia). *MALACOLOGIA-PHILADELPHIA-* , *48* (1/2), 265.

DUI	Mitochondria	Animals	*Meretrix Lamarckii*	Plazzi, F., Cassano, A., & Passamonti, M. (2015). The quest for D oubly U niparental I nheritance in heterodont bivalves and its detection in Meretrix lamarckii (Veneridae: Meretricinae). *Journal of Zoological Systematics and Evolutionary Research*, *53*(1), 87-94.
DUI	Mitochondria	Animals	*Modiolus modiolus Musculista senhousia*	Robicheau, B. M., Breton, S., & Stewart, D. T. (2017). Sequence motifs associated with paternal transmission of mitochondrial DNA in the horse mussel, Modiolus modiolus (Bivalvia: Mytilidae). *Gene*, *605*, 32-42.
DUI	Mitochondria	Animals	*Mytella charuana*	Alves, F. A., Beasley, C. R., Hoeh, W. R., da Rocha, R. M., Simone, L. R., & Tagliaro, C. H. (2012). Detection of mitochondrial DNA heteroplasmy suggests a doubly uniparental inheritance pattern in the mussel Mytella charruana. *Revista Brasileira de Biociências*, *10*(2), 176.
DUI	Mitochondria	Animals	*Mytilus californianus*	Beagley, C. T., Taylor, K. A., & Wolstenholme, D. R. (1997). Gender-associated diverse mitochondrial DNA molecules of the mussel Mytilus californianus. *Current genetics*, *31*(4), 318-324.
DUI	Mitochondria	Animals	*Mytilus coruscus*	Breton, S., Ghiselli, F., Passamonti, M., Milani, L., Stewart, D. T., & Hoeh, W. R. (2011). Evidence for a fourteenth mtDNA-encoded protein in the female-transmitted mtDNA of marine mussels (Bivalvia: Mytilidae). *PLoS One*, *6*(4), e19365.
DUI	Mitochondria	Animals	*Mytilus edulis*	Zouros, E., Ball, A. O., Saavedra, C., & Freeman, K. R. (1994). An unusual type of mitochondrial DNA inheritance in the blue mussel Mytilus. *Proceedings of the National Academy of Sciences*, *91*(16), 7463-7467.
DUI	Mitochondria	Animals	*Mytilus edulis* (blue mussel)	Skibinski, D. O., Gallagher, C., & Beynon, C. (1994). Sex-limited mitochondrial DNA transmission in the marine mussel Mytilus edulis. *Genetics*, *138*(3), 801-809.
DUI	Mitochondria	Animals	*Mytilus galloprovincialis*	Quesada, H., Skibinski, D. A., & Skibinski, D. O. (1996). Sex-biased heteroplasmy and mitochondrial DNA inheritance in the mussel Mytilus galloprovincialis Lmk. *Current genetics*, *29*(5), 423-426.
DUI	Mitochondria	Animals	*Mytilus trossulus*	Zouros, E., Ball, A. O., Saavedra, C., & Freeman, K. R. (1994). An unusual type of mitochondrial DNA inheritance in the blue mussel Mytilus. *Proceedings of the National Academy of Sciences*, *91*(16), 7463-7467.
DUI	Mitochondria	Animals	*Obliquaria reflexa*	Chapman, E. G., Piontkivska, H., Walker, J. M., Stewart, D. T., Curole, J. P., & Hoeh, W. R. (2008). Extreme primary and secondary protein structure variability in the chimeric male-transmitted cytochrome c oxidase subunit II protein in freshwater mussels: Evidence for an elevated amino acid substitution rate in the face of domain-specific purifying selection. *BMC Evolutionary Biology*, *8*(1), 1-16.
DUI	Mitochondria	Animals	*Obovaria olivaria*	Walker, J. M., Curole, J. P., Wade, D. E., Chapman, E. G., Bogan, A. E., Watters, G. T., & Hoeh, W. R. (2006). Taxonomic distribution and phylogenetic utility of gender-associated mitochondrial genomes in the Unionoida (Bivalvia). *MALACOLOGIA-PHILADELPHIA-*, *48*(1/2), 265.
DUI	Mitochondria	Animals	*Perumytilus purpuratus*	Vargas, J., Pérez, M., Toro, J., & Astorga, M. P. (2015). Presence of two mitochondrial genomes in the mytilid Perumytilus purpuratus: phylogenetic evidence for doubly uniparental inheritance. *Genetics and molecular biology*, *38*, 173-181.
DUI	Mitochondria	Animals	*Plectomerus dombeyanus*	Curole, J. P., & Kocher, T. D. (2005). Evolution of a unique mitotype-specific protein-coding extension of the cytochrome c oxidase II gene in freshwater mussels (Bivalvia: Unionoida). *Journal of Molecular Evolution*, *61*(3), 381-389.

DUI	Mitochondria	Animals	*Pleurobema sintoxia*	Chapman, E. G., Piontkivska, H., Walker, J. M., Stewart, D. T., Curole, J. P., & Hoeh, W. R. (2008). Extreme primary and secondary protein structure variability in the chimeric male-transmitted cytochrome c oxidase subunit II protein in freshwater mussels: Evidence for an elevated amino acid substitution rate in the face of domain-specific purifying selection. *BMC Evolutionary Biology* , *8* (1), 1-16.
DUI	Mitochondria	Animals	*Popenaias popeii*	Walker, J. M., Curole, J. P., Wade, D. E., Chapman, E. G., Bogan, A. E., Watters, G. T., & Hoeh, W. R. (2006). Taxonomic distribution and phylogenetic utility of gender-associated mitochondrial genomes in the Unionoida (Bivalvia). *MALACOLOGIA-PHILADELPHIA-* , *48* (1/2), 265.
DUI	Mitochondria	Animals	*Potamilus alatus*	Walker, J. M., Curole, J. P., Wade, D. E., Chapman, E. G., Bogan, A. E., Watters, G. T., & Hoeh, W. R. (2006). Taxonomic distribution and phylogenetic utility of gender-associated mitochondrial genomes in the Unionoida (Bivalvia). *MALACOLOGIA-PHILADELPHIA-* , *48* (1/2), 265.
DUI	Mitochondria	Animals	*Potamilus capax*	Walker, J. M., Curole, J. P., Wade, D. E., Chapman, E. G., Bogan, A. E., Watters, G. T., & Hoeh, W. R. (2006). Taxonomic distribution and phylogenetic utility of gender-associated mitochondrial genomes in the Unionoida (Bivalvia). *MALACOLOGIA-PHILADELPHIA-* , *48* (1/2), 265.
DUI	Mitochondria	Animals	*Potamilus ohiensis*	Walker, J. M., Curole, J. P., Wade, D. E., Chapman, E. G., Bogan, A. E., Watters, G. T., & Hoeh, W. R. (2006). Taxonomic distribution and phylogenetic utility of gender-associated mitochondrial genomes in the Unionoida (Bivalvia). *MALACOLOGIA-PHILADELPHIA-* , *48* (1/2), 265.
DUI	Mitochondria	Animals	*Potamilus purpuratus*	Hoeh, W. R., Stewart, D. T., & Guttman, S. I. (2002). High fidelity of mitochondrial genome transmission under the doubly uniparental mode of inheritance in freshwater mussels (Bivalvia: Unionoidea). *Evolution* , *56* (11), 2252-2261.
DUI	Mitochondria	Animals	*Pseudocardium sachalinense*	Plazzi, F. (2015). The detection of sex-linked heteroplasmy in Pseudocardium sachalinense (Bivalvia: Mactridae) and its implications for the distribution of doubly uniparental inheritance of mitochondrial DNA. *Journal of Zoological Systematics and Evolutionary Research* , *53* (3), 205-210.
DUI	Mitochondria	Animals	*Pseudodon vondembuschianus*	Walker, J. M., Curole, J. P., Wade, D. E., Chapman, E. G., Bogan, A. E., Watters, G. T., & Hoeh, W. R. (2006). Taxonomic distribution and phylogenetic utility of gender-associated mitochondrial genomes in the Unionoida (Bivalvia). *MALACOLOGIA-PHILADELPHIA-* , *48* (1/2), 265.
DUI	Mitochondria	Animals	*Ptychobranchus fasciolare*	Walker, J. M., Curole, J. P., Wade, D. E., Chapman, E. G., Bogan, A. E., Watters, G. T., & Hoeh, W. R. (2006). Taxonomic distribution and phylogenetic utility of gender-associated mitochondrial genomes in the Unionoida (Bivalvia). *MALACOLOGIA-PHILADELPHIA-* , *48* (1/2), 265.
DUI	Mitochondria	Animals	*Pyganodon fragilis*	Hoeh, W. R., Stewart, D. T., Sutherland, B. W., & Zouros, E. (1996). Multiple origins of gender-associated mitochondrial DNA lineages in bivalves (Mollusca: Bivalvia). *Evolution* , *50* (6), 2276-2286.
DUI	Mitochondria	Animals	*Pyganodon grandis*	Liu, H. P., Mitton, J. B., & Wu, S. K. (1996). Paternal mitochondrial DNA differentiation far exceeds maternal mitochondrial DNA and allozyme differentiation in the freshwater mussel, Anodonta grandis grandis. *Evolution* , 952-957.

DUI	Mitochondria	Animals	*Quadrula quadrula*	Curole, J. P., & Kocher, T. D. (2002). Ancient sex-specific extension of the cytochrome c oxidase II gene in bivalves and the fidelity of doubly-uniparental inheritance. *Molecular Biology and Evolution*, *19*(8), 1323-1328.
DUI	Mitochondria	Animals	*Quadrula refulgens*	Curole, J. P., & Kocher, T. D. (2002). Ancient sex-specific extension of the cytochrome c oxidase II gene in bivalves and the fidelity of doubly-uniparental inheritance. *Molecular Biology and Evolution*, *19*(8), 1323-1328.
DUI	Mitochondria	Animals	*Ruditapes philippinarum*	Ghiselli, F., Milani, L., Chang, P. L., Hedgecock, D., Davis, J. P., Nuzhdin, S. V., & Passamonti, M. (2012). De novo assembly of the Manila clam Ruditapes philippinarum transcriptome provides new insights into expression bias, mitochondrial doubly uniparental inheritance and sex determination. *Molecular biology and evolution*, *29*(2), 771-786.
DUI	Mitochondria	Animals	*Scrobicularia plana*	Gusman, A., Lecomte, S., Stewart, D. T., Passamonti, M., & Breton, S. (2016). Pursuing the quest for better understanding the taxonomic distribution of the system of doubly uniparental inheritance of mtDNA. *PeerJ*, *4*, e2760.
DUI	Mitochondria	Animals	*Scrobicularia plana*	Capt, C., Bouvet, K., Guerra, D., Robicheau, B. M., Stewart, D. T., Pante, E., & Breton, S. (2020). Unorthodox features in two venerid bivalves with doubly uniparental inheritance of mitochondria. *Scientific reports*, *10*(1), 1-13.
DUI	Mitochondria	Animals	*Semimytilus algosus*	Lubośny, M., Przyłucka, A., Śmietanka, B., & Burzyński, A. (2020). Semimytilus algosus: first known hermaphroditic mussel with doubly uniparental inheritance of mitochondrial DNA. *Scientific reports*, *10*(1), 1-12.
DUI	Mitochondria	Animals	*Solen grandis*	Lucentini, L., Plazzi, F., Sfriso, A. A., Pizzirani, C., Sfriso, A., & Chiesa, S. (2020). Additional taxonomic coverage of the doubly uniparental inheritance in bivalves: Evidence of sex-linked heteroplasmy in the razor clam Solen marginatus Pulteney, 1799, but not in the lagoon cockle Cerastoderma glaucum (Bruguière, 1789). *Journal of Zoological Systematics and Evolutionary Research*, *58*(2), 561-570.
DUI	Mitochondria	Animals	*Solenaia carinatus*	Huang, X. C., Rong, J., Liu, Y., Zhang, M. H., Wan, Y., Ouyang, S., ... & Wu, X. P. (2013). The complete maternally and paternally inherited mitochondrial genomes of the endangered freshwater mussel Solenaia carinatus (Bivalvia: Unionidae) and implications for Unionidae taxonomy. *PLoS One*, *8*(12), e84352.
DUI	Mitochondria	Animals	*Strophitus undulatus*	Stewart, D. T., Hoeh, W. R., Bauer, G., & Breton, S. (2013). Mitochondrial genes, sex determination and hermaphroditism in freshwater mussels (Bivalvia: Unionoida). In *Evolutionary biology: exobiology and evolutionary mechanisms* (pp. 245-255). Springer, Berlin, Heidelberg.
DUI	Mitochondria	Animals	*Toxolasma glans*	Stewart, D. T., Hoeh, W. R., Bauer, G., & Breton, S. (2013). Mitochondrial genes, sex determination and hermaphroditism in freshwater mussels (Bivalvia: Unionoida). In *Evolutionary biology: exobiology and evolutionary mechanisms* (pp. 245-255). Springer, Berlin, Heidelberg.
DUI	Mitochondria	Animals	*Toxolasma lividus*	Stewart, D. T., Hoeh, W. R., Bauer, G., & Breton, S. (2013). Mitochondrial genes, sex determination and hermaphroditism in freshwater mussels (Bivalvia: Unionoida). In *Evolutionary biology: exobiology and evolutionary mechanisms* (pp. 245-255). Springer, Berlin, Heidelberg.

DUI	Mitochondria	Animals	*Toxolasma minor*	Stewart, D. T., Hoeh, W. R., Bauer, G., & Breton, S. (2013). Mitochondrial genes, sex determination and hermaphroditism in freshwater mussels (Bivalvia: Unionoida). In *Evolutionary biology: exobiology and evolutionary mechanisms* (pp. 245-255). Springer, Berlin, Heidelberg.
DUI	Mitochondria	Animals	*Toxolasma paulus*	Stewart, D. T., Hoeh, W. R., Bauer, G., & Breton, S. (2013). Mitochondrial genes, sex determination and hermaphroditism in freshwater mussels (Bivalvia: Unionoida). In *Evolutionary biology: exobiology and evolutionary mechanisms* (pp. 245-255). Springer, Berlin, Heidelberg.
DUI	Mitochondria	Animals	*Truncilla truncate*	Walker, J. M., Curole, J. P., Wade, D. E., Chapman, E. G., Bogan, A. E., Watters, G. T., & Hoeh, W. R. (2006). Taxonomic distribution and phylogenetic utility of gender-associated mitochondrial genomes in the Unionoida (Bivalvia). *MALACOLOGIA-PHILADELPHIA-*, *48*(1/2), 265.
DUI	Mitochondria	Animals	*Unio crassus*	Soroka, M. (2010). Characteristics of mitochondrial DNA of unionid bivalves (Mollusca: Bivalvia: Unionidae). I. Detection and characteristics of doubly uniparental inheritance (DUI) of unionid mitochondrial DNA. *Folia malacologica*, *18*(4).
DUI	Mitochondria	Animals	*Unio delphinus*	Machordom, A., Araujo, R., Toledo, C., Zouros, E., & Ladoukakis, E. D. (2015). Female-dependent transmission of paternal mtDNA is a shared feature of bivalve species with doubly uniparental inheritance (DUI) of mitochondrial DNA. *Journal of Zoological Systematics and Evolutionary Research*, *53*(3), 200-204.
DUI	Mitochondria	Animals	*Unio pictorum*	Soroka, M. (2010). Characteristics of mitochondrial DNA of unionid bivalves (Mollusca: Bivalvia: Unionidae). I. Detection and characteristics of doubly uniparental inheritance (DUI) of unionid mitochondrial DNA. *Folia malacologica*, *18*(4).
DUI	Mitochondria	Animals	*Unio tumidus*	Soroka, M. (2010). Characteristics of mitochondrial DNA of unionid bivalves (Mollusca: Bivalvia: Unionidae). I. Detection and characteristics of doubly uniparental inheritance (DUI) of unionid mitochondrial DNA. *Folia malacologica*, *18*(4).
DUI	Mitochondria	Animals	*Utterbackia peggyae*	Breton, S., Stewart, D. T., Shepardson, S., Trdan, R. J., Bogan, A. E., Chapman, E. G., ... & Hoeh, W. R. (2011). Novel protein genes in animal mtDNA: a new sex determination system in freshwater mussels (Bivalvia: Unionoida)?. *Molecular Biology and Evolution*, *28*(5), 1645-1659.
DUI	Mitochondria	Animals	*Utterbackia peninsularis*	Breton, S., Stewart, D. T., Shepardson, S., Trdan, R. J., Bogan, A. E., Chapman, E. G., ... & Hoeh, W. R. (2011). Novel protein genes in animal mtDNA: a new sex determination system in freshwater mussels (Bivalvia: Unionoida)?. *Molecular Biology and Evolution*, *28*(5), 1645-1659.
DUI	Mitochondria	Animals	*Venerupis philippinarum*	Passamonti, M., & Scali, V. (2001). Gender-associated mitochondrial DNA heteroplasmy in the venerid clam Tapes philippinarum (Mollusca Bivalvia). *Current genetics*, *39*(2), 117-124.
DUI	Mitochondria	Animals	*Venustaconcha ellipsiformis*	Chakrabarti, R., Walker, J. M., Stewart, D. T., Trdan, R. J., Vijayaraghavan, S., Curole, J. P., & Hoeh, W. R. (2006). Presence of a unique male-specific extension of C-terminus to the cytochrome c oxidase subunit II protein coded by the male-transmitted mitochondrial genome of Venustaconcha ellipsiformis (Bivalvia: Unionoidea). *FEBS letters*, *580*(3), 862-866.
DUI	Mitochondria	Animals	*Villosa lienosa*	Curole, J. P., & Kocher, T. D. (2005). Evolution of a unique mitotype-specific protein-coding extension of the cytochrome c oxidase II gene in freshwater mussels (Bivalvia: Unionoida). *Journal of Molecular Evolution*, *61*(3), 381-389.

DUI	Mitochondria	Animals	*Villosa villosa*	Walker, J. M., Curole, J. P., Wade, D. E., Chapman, E. G., Bogan, A. E., Watters, G. T., & Hoeh, W. R. (2006). Taxonomic distribution and phylogenetic utility of gender-associated mitochondrial genomes in the Unionoida (Bivalvia). *MALACOLOGIA-PHILADELPHIA-*, *48*(1/2), 265.
DUI	Mitochondria	Animals	*Yoldia hyperborea*	Gusman, A., Lecomte, S., Stewart, D. T., Passamonti, M., & Breton, S. (2016). Pursuing the quest for better understanding the taxonomic distribution of the system of doubly uniparental inheritance of mtDNA. *PeerJ*, *4*, e2760.
Paternal Inheritance	Mitochondria	Animals	*Antheraea proylei*	Arunkumar, K. P., Metta, M., & Nagaraju, J. (2006). Molecular phylogeny of silkmoths reveals the origin of domesticated silkmoth, Bombyx mori from Chinese Bombyx mandarina and paternal inheritance of Antheraea proylei mitochondrial DNA. *Molecular Phylogenetics and Evolution*, *40*(2), 419-427.
Paternal Leakage	Mitochondria	Animals	*Alectoris graeca* (rock partridge)	Gandolfi, A., Crestanello, B., Fagotti, A., Simoncelli, F., Chiesa, S., Girardi, M., ... & Lucentini, L. (2017). New evidences of mitochondrial DNA heteroplasmy by putative paternal leakage between the rock partridge (Alectoris graeca) and the chukar partridge (Alectoris chukar). *PLoS One*, *12*(1), e0170507.
Paternal Leakage	Mitochondria	Animals	*Apis mellifera* (honeybee)	Meusel, M. S., & Moritz, R. F. (1993). Transfer of paternal mitochondrial DNA during fertilization of honeybee (Apis mellifera L.) eggs. *Current genetics*, *24*(6), 539-543.
Paternal Leakage	Mitochondria	Animals	*Bos taurus* (cow)	Steinborn, R., Zakhartchenko, V., Jelyazkov, J., Klein, D., Wolf, E., Müller, M., & Brem, G. (1998). Composition of parental mitochondrial DNA in cloned bovine embryos. *FEBS letters*, *426*(3), 352-356.
Paternal Leakage	Mitochondria	Animals	*Buthus mardoechi*	Gantenbein, B., Fet, V., Gantenbein-Ritter, I. A., & Balloux, F. (2005). Evidence for recombination in scorpion mitochondrial DNA (Scorpiones: Buthidae). *Proceedings of the Royal Society B: Biological Sciences*, *272*(1564), 697-704.
Paternal Leakage	Mitochondria	Animals	*Caenorhabditis briggsae*	Adineh, S., & Ross, J. A. (2019). The ebb and flow of heteroplasmy during intra-species hybridization in Caenorhabditis briggsae. *BioRxiv*, 623207.
Paternal Leakage	Mitochondria	Animals	*Chlamydosaurus kingii*	Ujvari, B., Dowton, M., & Madsen, T. (2007). Mitochondrial DNA recombination in a free-ranging Australian lizard. *Biology Letters*, *3*(2), 189-192.
Paternal Leakage	Mitochondria	Animals	*Cimex lectularius* (bed bug)	Robison, G. A., Balvin, O., Schal, C., Vargo, E. L., & Booth, W. (2015). Extensive mitochondrial heteroplasmy in natural populations of a resurging human pest, the bed bug (Hemiptera: Cimicidae). *Journal of medical entomology*, *52*(4), 734-738.
Paternal Leakage	Mitochondria	Animals	*Cyprinus carpio haematopterus x Megalobrama amblycephala*	Wang, Y., Sun, W., Gu, Q., Yao, J., Tan, H., Huang, X., ... & Liu, S. (2021). Variations in the Mitochondrial Genome of a Goldfish-Like Hybrid [Koi Carp (♀)× Blunt Snout Bream (♂)] Indicate Paternal Leakage. *Frontiers in genetics*, *11*, 1688.
Paternal Leakage	Mitochondria	Animals	*Drosophila melanogaster*	Sherengul, W., Kondo, R., & Matsuura, E. T. (2006). Analysis of paternal transmission of mitochondrial DNA in Drosophila. *Genes & genetic systems*, *81*(6), 399-404.
Paternal Leakage	Mitochondria	Animals	*Drosophila mauritiana* x *D. simulans* hybrid	Kondo, R., Satta, Y., Matsuura, E. T., Ishiwa, H., Takahata, N., & Chigusa, S. I. (1990). Incomplete maternal transmission of mitochondrial DNA in Drosophila. *Genetics*, *126*(3), 657-663.
Paternal Leakage	Mitochondria	Animals	*Drosophila simulans*	Kondo, R., Matsuura, E. T., & Chigusa, S. I. (1992). Further observation of paternal transmission of Drosophila mitochondrial DNA by PCR selective amplification method. *Genetics Research*, *59*(2), 81-84.

Paternal Leakage	Mitochondria	Animals	*Drosophila simulans*	Wolff, J. N., Nafisinia, M., Sutovsky, P., & Ballard, J. W. O. (2013). Paternal transmission of mitochondrial DNA as an integral part of mitochondrial inheritance in metapopulations of Drosophila simulans. *Heredity*, *110*(1), 57-62.
Paternal Leakage	Mitochondria	Animals	*Drosophila simulans* x *D. mauritiana* hybrid	Polovina, E. S., Parakatselaki, M. E., & Ladoukakis, E. D. (2020). Paternal leakage of mitochondrial DNA and maternal inheritance of heteroplasmy in Drosophila hybrids. *Scientific Reports*, *10*(1), 1-9.
Paternal Leakage	Mitochondria	Animals	*Drosophila simulans* x *D. sec hellia* hybrid	Dokianakis, E., & Ladoukakis, E. D. (2014). Different degree of paternal mt DNA leakage between male and female progeny in interspecific D rosophila crosses. *Ecology and evolution*, *4*(13), 2633-2641.
Paternal Leakage	Mitochondria	Animals	*Drosophila teissieri* x *D. mau ritiana* hybrid	Dokianakis, E., & Ladoukakis, E. D. (2014). Different degree of paternal mt DNA leakage between male and female progeny in interspecific D rosophila crosses. *Ecology and evolution*, *4*(13), 2633-2641.
Paternal Leakage	Mitochondria	Animals	*Drosophila yakuba* x *D. mauritiana* hybrid	Dokianakis, E., & Ladoukakis, E. D. (2014). Different degree of paternal mt DNA leakage between male and female progeny in interspecific D rosophila crosses. *Ecology and evolution*, *4*(13), 2633-2641.
Paternal Leakage	Mitochondria	Animals	*Gallus gallus*	Alexander M, Ho SY, Molak M, Barnett R, Carlborg Ö, Dorshorst B, et al. Mitogenomic analysis of a 50-generation chicken pedigree reveals a rapid rate of mitochondrial evolution and evidence for paternal mtDNA inheritance. Biol Lett. 2015; 11: 20150561. pmid:26510672
Paternal Leakage	Mitochondria	Animals	*Heliothis virescens* x *H.subflexa* (budworm hybrid)	Lansman, R. A., Avise, J. C., & Huettel, M. D. (1983). Critical experimental test of the possibility of" paternal leakage" of mitochondrial DNA. *Proceedings of the National Academy of Sciences*, *80*(7), 1969-1971.
Paternal Leakage	Mitochondria	Animals	*Homo sapiens* (human)	Schwartz, M., & Vissing, J. (2002). Paternal inheritance of mitochondrial DNA. *New England Journal of Medicine*, *347*(8), 576-580.
Paternal Leakage	Mitochondria	Animals	*Homo sapiens* (human)	Luo, S., Valencia, C. A., Zhang, J., Lee, N. C., Slone, J., Gui, B., ... & Huang, T. (2018). Biparental inheritance of mitochondrial DNA in humans. *Proceedings of the National Academy of Sciences*, *115*(51), 13039-13044.
Paternal Leakage	Mitochondria	Animals	*Magicicada septendecim; M. septendecula; M. cassini* (17-year period cicada hybrids)	Fontaine, K. M., Cooley, J. R., & Simon, C. (2007). Evidence for paternal leakage in hybrid periodical cicadas (Hemiptera: Magicicada spp.). *PloS one*, *2*(9), e892.
Paternal Leakage	Mitochondria	Animals	*Mesobuthus caucasius*	Gantenbein, B., Fet, V., Gantenbein-Ritter, I. A., & Balloux, F. (2005). Evidence for recombination in scorpion mitochondrial DNA (Scorpiones: Buthidae). *Proceedings of the Royal Society B: Biological Sciences*, *272*(1564), 697-704.
Paternal Leakage	Mitochondria	Animals	*Mesobuthus eupeus*	Gantenbein, B., Fet, V., Gantenbein-Ritter, I. A., & Balloux, F. (2005). Evidence for recombination in scorpion mitochondrial DNA (Scorpiones: Buthidae). *Proceedings of the Royal Society B: Biological Sciences*, *272*(1564), 697-704.
Paternal Leakage	Mitochondria	Animals	*Mesobuthus gibbosus*	Gantenbein, B., Fet, V., Gantenbein-Ritter, I. A., & Balloux, F. (2005). Evidence for recombination in scorpion mitochondrial DNA (Scorpiones: Buthidae). *Proceedings of the Royal Society B: Biological Sciences*, *272*(1564), 697-704.
Paternal Leakage	Mitochondria	Animals	*Mus musculus* (mouse)	Gyllensten, U., Wharton, D., Josefsson, A., & Wilson, A. C. (1991). Paternal inheritance of mitochondrial DNA in mice. *Nature*, *352*(6332), 255-257.

Paternal Leakage	Mitochondria	Animals	*Mus musculus* (mouse)	Shitara, H., Hayashi, J. I., Takahama, S., Kaneda, H., & Yonekawa, H. (1998). Maternal inheritance of mouse mtDNA in interspecific hybrids: segregation of the leaked paternal mtDNA followed by the prevention of subsequent paternal leakage. *Genetics*, *148*(2), 851-857.
Paternal Leakage	Mitochondria	Animals	*Nyctereutes procyonoides*	Nisztuk-Pacek, S., Ślaska, B., Grzybowska-Szatkowska, L., & Babicz, M. (2019). Paternal leakage of mitochondrial dna in the raccoon dog (Nyctereutes procyoNoides gray 1834). *Annals of Animal Science*, *19*(1), 61-69.
Paternal Leakage	Mitochondria	Animals	*Ovis aries* (sheep)	Zhao, X., Li, N., Guo, W., Hu, X., Liu, Z., Gong, G., ... & Wu, C. (2004). Further evidence for paternal inheritance of mitochondrial DNA in the sheep (Ovis aries). *Heredity*, *93*(4), 399-403.
Paternal Leakage	Mitochondria	Animals	*Papillo glaucus*	Andolfatto, P., Scriber, J. M., & Charlesworth, B. (2003). No association between mitochondrial DNA haplotypes and a female-limited mimicry phenotype in Papilio glaucus. *Evolution*, *57*(2), 305-316.
Paternal Leakage	Mitochondria	Animals	*Parus major* (great tit)	Kvist, L., Martens, J., Nazarenko, A. A., & Orell, M. (2003). Paternal leakage of mitochondrial DNA in the great tit (Parus major). *Molecular Biology and Evolution*, *20*(2), 243-247.
Paternal Leakage	Mitochondria	Animals	*Platichthys flesus*	Hoarau, G., Holla, S., Lescasse, R., Stam, W. T., & Olsen, J. L. (2002). Heteroplasmy and evidence for recombination in the mitochondrial control region of the flatfish Platichthys flesus. *Molecular Biology and Evolution*, *19*(12), 2261-2264.
Paternal Leakage	Mitochondria	Animals	*Rhipicephalus*	Mastrantonio, V., Latrofa, M. S., Porretta, D., Lia, R. P., Parisi, A., Iatta, R., ... & Urbanelli, S. (2019). Paternal leakage and mtDNA heteroplasmy in Rhipicephalus spp. ticks. *Scientific reports*, *9*(1), 1-8.
Paternal Leakage	Mitochondria	Animals	*Scomberomorus munroi* (spotted mackerel) x *Scomberomorus semifasciatus* (gray mackerel)	Morgan, J. A., Macbeth, M., Broderick, D., Whatmore, P., Street, R., Welch, D. J., & Ovenden, J. R. (2013). Hybridisation, paternal leakage and mitochondrial DNA linearization in three anomalous fish (Scombridae). *Mitochondrion*, *13*(6), 852-861.
Paternal Leakage	Mitochondria	Animals	*Thrips tabaci*	Gawande, S. J., Anandhan, S., Ingle, A. A., Jacobson, A., & Asokan, R. (2017). Heteroplasmy due to coexistence of mtCOI haplotypes from different lineages of the Thrips tabaci cryptic species group. *Bulletin of entomological research*, *107*(4), 534-542.
Divergent Heteroplasmy (unknown mechanism)	Mitochondria	Animals	*Gonioctena intermedia*	Kastally, C., & Mardulyn, P. (2017). Widespread co-occurrence of two distantly related mitochondrial genomes in individuals of the leaf beetle Gonioctena intermedia. *Biology letters*, *13*(11), 20170570.
Divergent Heteroplasmy (unknown mechanism)	Mitochondria	Animals	*Sphenodon punctatus* (tuatara)	Macey, J. R., Pabinger, S., Barbieri, C. G., Buring, E. S., Gonzalez, V. L., Mulcahy, D. G., ... & Gemmell, N. J. (2021). Evidence of two deeply divergent co-existing mitochondrial genomes in the Tuatara reveals an extremely complex genomic organization. *Communications biology*, *4*(1), 1-10.
Biparental Inheritance	Mitochondria	Fungi	*Saccharomyces cerevisiae* (Baker's yeast)	Birky Jr, C. W. (1978). Transmission genetics of mitochondria and chloroplasts. *Annual review of genetics*, *12*(1), 471-512.

Biparental Inheritance	Mitochondria	Fungi	*Cryptococcus neoformans*	Yan, Z., Sun, S., Shahid, M., & Xu, J. (2007). Environment factors can influence mitochondrial inheritance in the fungus Cryptococcus neoformans. *Fungal Genetics and Biology* , *44* (5), 315-322.
Biparental Inheritance	Chloroplasts	Plants	*Borago oficinalis*	Sears, B. B. (1980). Elimination of plastids during spermatogenesis and fertilization in the plant kingdom. *Plasmid* , *4* (3), 233-255.
Biparental Inheritance	Chloroplasts	Plants	*Campanula alliariafolia*	Corriveau, J. L., & Coleman, A. W. (1988). Rapid screening method to detect potential biparental inheritance of plastid DNA and results for over 200 angiosperm species. *American Journal of Botany* , *75* (10), 1443-1458.
Biparental Inheritance	Chloroplasts	Plants	*Campanula americana*	Barnard-Kubow, K. B., McCoy, M. A., & Galloway, L. F. (2017). Biparental chloroplast inheritance leads to rescue from cytonuclear incompatibility. *New Phytologist* , *213* (3), 1466-1476.
Biparental Inheritance	Chloroplasts	Plants	*Campanula carpatica*	Corriveau, J. L., & Coleman, A. W. (1988). Rapid screening method to detect potential biparental inheritance of plastid DNA and results for over 200 angiosperm species. *American Journal of Botany* , *75* (10), 1443-1458.
Biparental Inheritance	Chloroplasts	Plants	*Campanula rapunculoides*	Corriveau, J. L., & Coleman, A. W. (1988). Rapid screening method to detect potential biparental inheritance of plastid DNA and results for over 200 angiosperm species. *American Journal of Botany* , *75* (10), 1443-1458.
Biparental Inheritance	Chloroplasts	Plants	*Cryptomeria japonica* (sugi)	Ohba, K. (1971). Paternal transmission of a plastid anomaly in some reciprocal crosses of sugi, Cryptomeria japonica. *Silvae Genet.* , *20* , 101-107.
Biparental Inheritance	Chloroplasts	Plants	*Dionaea muscipula*	Corriveau, J. L., & Coleman, A. W. (1988). Rapid screening method to detect potential biparental inheritance of plastid DNA and results for over 200 angiosperm species. *American Journal of Botany* , *75* (10), 1443-1458.
Biparental Inheritance	Chloroplasts	Plants	*Drosera capillaris*	Corriveau, J. L., & Coleman, A. W. (1988). Rapid screening method to detect potential biparental inheritance of plastid DNA and results for over 200 angiosperm species. *American Journal of Botany* , *75* (10), 1443-1458.
Biparental Inheritance	Chloroplasts	Plants	*Fagopyrum esculentum*	Reboud, X., & Zeyl, C. (1994). Organelle inheritance in plants. *Heredity* , *72* (2), 132-140.
Biparental Inheritance	Chloroplasts	Plants	*Geranium bohemicum*	Sears, B. B. (1980). Elimination of plastids during spermatogenesis and fertilization in the plant kingdom. *Plasmid* , *4* (3), 233-255.
Biparental Inheritance	Chloroplasts	Plants	*Geranium bohemicum*	Sears, B. B. (1980). Elimination of plastids during spermatogenesis and fertilization in the plant kingdom. *Plasmid* , *4* (3), 233-255.
Biparental Inheritance	Chloroplasts	Plants	*Geranium maculatum*	Corriveau, J. L., & Coleman, A. W. (1988). Rapid screening method to detect potential biparental inheritance of plastid DNA and results for over 200 angiosperm species. *American Journal of Botany* , *75* (10), 1443-1458.
Biparental Inheritance	Chloroplasts	Plants	*Geranium pratense*	Corriveau, J. L., & Coleman, A. W. (1988). Rapid screening method to detect potential biparental inheritance of plastid DNA and results for over 200 angiosperm species. *American Journal of Botany* , *75* (10), 1443-1458.
Biparental Inheritance	Chloroplasts	Plants	*Geranium sanguineum*	Corriveau, J. L., & Coleman, A. W. (1988). Rapid screening method to detect potential biparental inheritance of plastid DNA and results for over 200 angiosperm species. *American Journal of Botany* , *75* (10), 1443-1458.
Biparental Inheritance	Chloroplasts	Plants	*Hypericum acutum*	Tilney-Basset, R. A. E. (1978). The inheritance and behaviour of plastids.

Biparental Inheritance	Chloroplasts	Plants	*Lathyrus japonicus*	Corriveau, J. L., & Coleman, A. W. (1988). Rapid screening method to detect potential biparental inheritance of plastid DNA and results for over 200 angiosperm species. *American Journal of Botany* , *75* (10), 1443-1458.
Biparental Inheritance	Chloroplasts	Plants	*Lathyrus odoratus*	Corriveau, J. L., & Coleman, A. W. (1988). Rapid screening method to detect potential biparental inheritance of plastid DNA and results for over 200 angiosperm species. *American Journal of Botany* , *75* (10), 1443-1458.
Biparental Inheritance	Chloroplasts	Plants	*Linnaea borealis*	Corriveau, J. L., & Coleman, A. W. (1988). Rapid screening method to detect potential biparental inheritance of plastid DNA and results for over 200 angiosperm species. *American Journal of Botany* , *75* (10), 1443-1458.
Biparental Inheritance	Chloroplasts	Plants	*Lobelia syphilitica*	Corriveau, J. L., & Coleman, A. W. (1988). Rapid screening method to detect potential biparental inheritance of plastid DNA and results for over 200 angiosperm species. *American Journal of Botany* , *75* (10), 1443-1458.
Biparental Inheritance	Chloroplasts	Plants	*Lonicera japonica*	Corriveau, J. L., & Coleman, A. W. (1988). Rapid screening method to detect potential biparental inheritance of plastid DNA and results for over 200 angiosperm species. *American Journal of Botany* , *75* (10), 1443-1458.
Biparental Inheritance	Chloroplasts	Plants	*Lpilobium hirsutum*	Reboud, X., & Zeyl, C. (1994). Organelle inheritance in plants. *Heredity* , *72* (2), 132-140.
Biparental Inheritance	Chloroplasts	Plants	*Medicago truncatula*	Sears, B. B. (1980). Elimination of plastids during spermatogenesis and fertilization in the plant kingdom. *Plasmid* , *4* (3), 233-255.
Biparental Inheritance	Chloroplasts	Plants	*Melilotus alba*	Corriveau, J. L., & Coleman, A. W. (1988). Rapid screening method to detect potential biparental inheritance of plastid DNA and results for over 200 angiosperm species. *American Journal of Botany* , *75* (10), 1443-1458.
Biparental Inheritance	Chloroplasts	Plants	*Melilotus indica*	Corriveau, J. L., & Coleman, A. W. (1988). Rapid screening method to detect potential biparental inheritance of plastid DNA and results for over 200 angiosperm species. *American Journal of Botany* , *75* (10), 1443-1458.
Biparental Inheritance	Chloroplasts	Plants	*Melilotus officinalis*	Corriveau, J. L., & Coleman, A. W. (1988). Rapid screening method to detect potential biparental inheritance of plastid DNA and results for over 200 angiosperm species. *American Journal of Botany* , *75* (10), 1443-1458.
Biparental Inheritance	Chloroplasts	Plants	*Nepeta cataria*	Sears, B. B. (1980). Elimination of plastids during spermatogenesis and fertilization in the plant kingdom. *Plasmid* , *4* (3), 233-255.
Biparental Inheritance	Chloroplasts	Plants	*Nepeta cataria*	Reboud, X., & Zeyl, C. (1994). Organelle inheritance in plants. *Heredity* , *72* (2), 132-140.
Biparental Inheritance	Chloroplasts	Plants	*Oenothera ammophila; Oe. perennis*	Corriveau, J. L., & Coleman, A. W. (1988). Rapid screening method to detect potential biparental inheritance of plastid DNA and results for over 200 angiosperm species. *American Journal of Botany* , *75* (10), 1443-1458.
Biparental Inheritance	Chloroplasts	Plants	*Oenothera biennis*	Corriveau, J. L., & Coleman, A. W. (1988). Rapid screening method to detect potential biparental inheritance of plastid DNA and results for over 200 angiosperm species. *American Journal of Botany* , *75* (10), 1443-1458.
Biparental Inheritance	Chloroplasts	Plants	*Oenothera grandiflora*	Corriveau, J. L., & Coleman, A. W. (1988). Rapid screening method to detect potential biparental inheritance of plastid DNA and results for over 200 angiosperm species. *American Journal of Botany* , *75* (10), 1443-1458.

Biparental Inheritance	Chloroplasts	Plants	*Oenothera hookeri; Oe. suaveolens; Oe. lamarckiaria; Oe. atrovirens* (hybrids)	Chiu, W. L., Stubbe, W., & Sears, B. B. (1988). Plastid inheritance in Oenothera: organelle genome modifies the extent of biparental plastid transmission. *Current genetics*, *13*(2), 181-189.
Biparental Inheritance	Chloroplasts	Plants	*Oenothera macrosceles*	Corriveau, J. L., & Coleman, A. W. (1988). Rapid screening method to detect potential biparental inheritance of plastid DNA and results for over 200 angiosperm species. *American Journal of Botany*, *75*(10), 1443-1458.
Biparental Inheritance	Chloroplasts	Plants	*Passiflora edulis*	Corriveau, J. L., & Coleman, A. W. (1988). Rapid screening method to detect potential biparental inheritance of plastid DNA and results for over 200 angiosperm species. *American Journal of Botany*, *75*(10), 1443-1458.
Biparental Inheritance	Chloroplasts	Plants	*Pelargonium peltatum*	Corriveau, J. L., & Coleman, A. W. (1988). Rapid screening method to detect potential biparental inheritance of plastid DNA and results for over 200 angiosperm species. *American Journal of Botany*, *75*(10), 1443-1458.
Biparental Inheritance	Chloroplasts	Plants	*Pelargonium zonale; P. inquinans* (hybrids)	Weihe, A., Apitz, J., Pohlheim, F., Salinas-Hartwig, A., & Börner, T. (2009). Biparental inheritance of plastidial and mitochondrial DNA and hybrid variegation in Pelargonium. *Molecular Genetics and Genomics*, *282*(6), 587-593.
Biparental Inheritance	Chloroplasts	Plants	*Pennisetum americanum*	Rao, M. K., & Koduru, P. R. K. (1978). Cytogenetics of a factor for syncyte formation and male sterility in Pennisetum americanum. *Theoretical and Applied Genetics*, *53*(1), 1-7.
Biparental Inheritance	Chloroplasts	Plants	*Phaseolus vulgaris*	Sears, B. B. (1980). Elimination of plastids during spermatogenesis and fertilization in the plant kingdom. *Plasmid*, *4*(3), 233-255.
Biparental Inheritance	Chloroplasts	Plants	*Pinus massoniana*	Ni, Z., Zhou, P., Xin, Y., Xu, M., & Xu, L. A. (2021). Parent–offspring variation transmission in full-sib families revealed predominantly paternal inheritance of chloroplast DNA in Pinus massoniana (Pinaceae). *Tree Genetics & Genomes*, *17*(4), 1-7.
Biparental Inheritance	Chloroplasts	Plants	*Platycodon grandiflorum*	Corriveau, J. L., & Coleman, A. W. (1988). Rapid screening method to detect potential biparental inheritance of plastid DNA and results for over 200 angiosperm species. *American Journal of Botany*, *75*(10), 1443-1458.
Biparental Inheritance	Chloroplasts	Plants	*Plumbago auriculata*	Corriveau, J. L., & Coleman, A. W. (1988). Rapid screening method to detect potential biparental inheritance of plastid DNA and results for over 200 angiosperm species. *American Journal of Botany*, *75*(10), 1443-1458.
Biparental Inheritance	Chloroplasts	Plants	*Plumbago capensis*	Corriveau, J. L., & Coleman, A. W. (1988). Rapid screening method to detect potential biparental inheritance of plastid DNA and results for over 200 angiosperm species. *American Journal of Botany*, *75*(10), 1443-1458.
Biparental Inheritance	Chloroplasts	Plants	*Plumbago larpentae*	Corriveau, J. L., & Coleman, A. W. (1988). Rapid screening method to detect potential biparental inheritance of plastid DNA and results for over 200 angiosperm species. *American Journal of Botany*, *75*(10), 1443-1458.
Biparental Inheritance	Chloroplasts	Plants	*Plumbago zeylanica*	Corriveau, J. L., & Coleman, A. W. (1988). Rapid screening method to detect potential biparental inheritance of plastid DNA and results for over 200 angiosperm species. *American Journal of Botany*, *75*(10), 1443-1458.
Biparental Inheritance	Chloroplasts	Plants	*Rhipsalidopsisg aertneri*	Corriveau, J. L., & Coleman, A. W. (1988). Rapid screening method to detect potential biparental inheritance of plastid DNA and results for over 200 angiosperm species. *American Journal of Botany*, *75*(10), 1443-1458.
Biparental Inheritance	Chloroplasts	Plants	*Rhododendron*	Tilney-Basset, R. A. E. (1978). The inheritance and behaviour of plastids.

Biparental Inheritance	Chloroplasts	Plants	*Rhododendronm aximum*	Corriveau, J. L., & Coleman, A. W. (1988). Rapid screening method to detect potential biparental inheritance of plastid DNA and results for over 200 angiosperm species. *American Journal of Botany*, *75*(10), 1443-1458.
Biparental Inheritance	Chloroplasts	Plants	*Secale cereale* (rye)	Sears, B. B. (1980). Elimination of plastids during spermatogenesis and fertilization in the plant kingdom. *Plasmid*, *4*(3), 233-255.
Biparental Inheritance	Chloroplasts	Plants	Silene otites	Tilney-Basset, R. A. E. (1978). The inheritance and behaviour of plastids.
Biparental Inheritance	Chloroplasts	Plants	*Silene pseudotites*	Sears, B. B. (1980). Elimination of plastids during spermatogenesis and fertilization in the plant kingdom. *Plasmid*, *4*(3), 233-255.
Biparental Inheritance	Chloroplasts	Plants	*Strelitzia reginae*	Corriveau, J. L., & Coleman, A. W. (1988). Rapid screening method to detect potential biparental inheritance of plastid DNA and results for over 200 angiosperm species. *American Journal of Botany*, *75*(10), 1443-1458.
Biparental Inheritance	Chloroplasts	Plants	*Wisteria sinensis*	Corriveau, J. L., & Coleman, A. W. (1988). Rapid screening method to detect potential biparental inheritance of plastid DNA and results for over 200 angiosperm species. *American Journal of Botany*, *75*(10), 1443-1458.
Biparental Inheritance	Chloroplasts	Plants	*Zygocactus truncatus*	Corriveau, J. L., & Coleman, A. W. (1988). Rapid screening method to detect potential biparental inheritance of plastid DNA and results for over 200 angiosperm species. *American Journal of Botany*, *75*(10), 1443-1458.
Biparental Inheritance	Mitochondria	Plants	*Pelargonium zonale; P. inquinans* (hybrids)	Weihe, A., Apitz, J., Pohlheim, F., Salinas-Hartwig, A., & Börner, T. (2009). Biparental inheritance of plastidial and mitochondrial DNA and hybrid variegation in Pelargonium. *Molecular Genetics and Genomics*, *282*(6), 587-593.
Maternal Leakage	Chloroplasts	Plants	*Larix* (larch) hybrids	Szmidt, A. E., Aldén, T., & Hällgren, J. E. (1987). Paternal inheritance of chloroplast DNA in Larix. *Plant Molecular Biology*, *9*(1), 59-64.
Maternal Leakage	Chloroplasts	Plants	*Larix leptolepis; L. decidua; L. gmelini; L. siberica; L. olgensis; L. laricina* (larch hybrids)	Cipriani, G., Testolin, R., & Morgante, M. (1995). Paternal inheritance of plastids in interspecific hybrids of the genus Actinidia revealed by PCR-amplification of chloroplast DNA fragments. *Molecular and General Genetics MGG*, *247*(6), 693-697.
Maternal Leakage	Chloroplasts	Plants	*Pinus radiata* (Monterey pine)	Cato, S. A., & Richardson, T. E. (1996). Inter-and intraspecific polymorphism at chloroplast SSR loci and the inheritance of plastids in Pinus radiata D. Don. *Theoretical and Applied Genetics*, *93*(4), 587-592.
Paternal Inheritance	Chloroplasts	Plants	*Actinidia arguta; A. deliciosa; A. kolomikta; A. chinensis* (kiwifruit hybrids)	Cipriani, G., Testolin, R., & Morgante, M. (1995). Paternal inheritance of plastids in interspecific hybrids of the genus Actinidia revealed by PCR-amplification of chloroplast DNA fragments. *Molecular and General Genetics MGG*, *247*(6), 693-697.
Paternal Inheritance	Chloroplasts	Plants	*Biota orientalis* (oriental arborvitae)	Chesnoy, L. (1969). On the origin of cytoplasm of embryos in Biota orientalis Endl.(Cupressaceae). *Acad Sci Compt Rend Ser D*.
Paternal Inheritance	Chloroplasts	Plants	*Calocedrus decurrens* (incense cedar)	Neale, D. B., Marshall, K. A., & Harry, D. E. (1991). Inheritance of chloroplast and mitochondrial DNA in incense-cedar (Calocedrus decurrens). *Canadian Journal of Forest Research*, *21*(5), 717-720.
Paternal Inheritance	Chloroplasts	Plants	*Daucus carota; D. maximus; D. muricatus (wild carrot)*	Boblenz, K., Nothnagel, T., & Metzlaff, M. (1990). Paternal inheritance of plastids in the genus Daucus. *Molecular and General Genetics MGG*, *220*(3), 489-491.

Paternal Inheritance	Chloroplasts	Plants	*Larix* (larch) hybrids	Szmidt, A. E., Aldén, T., & Hällgren, J. E. (1987). Paternal inheritance of chloroplast DNA in Larix. *Plant Molecular Biology*, *9*(1), 59-64.
Paternal Inheritance	Chloroplasts	Plants	*Larix leptolepis; L. decidua; L. gmelini; L. siberica; L. olgensis; L. laricina* (larch hybrids)	Cipriani, G., Testolin, R., & Morgante, M. (1995). Paternal inheritance of plastids in interspecific hybrids of the genus Actinidia revealed by PCR-amplification of chloroplast DNA fragments. *Molecular and General Genetics MGG*, *247*(6), 693-697.
Paternal Inheritance	Chloroplasts	Plants	*Medicago sativa* (alfalfa)	Schumann, C. M., & Hancock, J. F. (1989). Paternal inheritance of plastids in Medicago sativa. *Theoretical and Applied Genetics*, *78*(6), 863-866.
Paternal Inheritance	Chloroplasts	Plants	*Petunia hybrida (garden petunia)*	Cornu, A., & Dulieu, H. (1988). Pollen Transmission of Plastid–DNA Under Genotypic Control in Petunia hybrida Hort. *Journal of Heredity*, *79*(1), 40-44.
Paternal Inheritance	Chloroplasts	Plants	*Picea glauca* (white spruce)	Stine, M., Sears, B. B., & Keathley, D. E. (1989). Inheritance of plastids in interspecific hybrids of blue spruce and white spruce. *Theoretical and applied genetics*, *78*(6), 768-774.
Paternal Inheritance	Chloroplasts	Plants	*Picea pungens* (blue spruce)	Stine, M., Sears, B. B., & Keathley, D. E. (1989). Inheritance of plastids in interspecific hybrids of blue spruce and white spruce. *Theoretical and applied genetics*, *78*(6), 768-774.
Paternal Inheritance	Chloroplasts	Plants	*Pinus monticola* (western white pine)	White, E. E. (1990). Chloroplast DNA in Pinus monticola. *Theoretical and Applied Genetics*, *79*(1), 119-124.
Paternal Inheritance	Chloroplasts	Plants	*Pinus radiata* (Monterey pine)	Cato, S. A., & Richardson, T. E. (1996).Z Inter-and intraspecific polymorphism at chloroplast SSR loci and the inheritance of plastids in Pinus radiata D. Don. *Theoretical and Applied Genetics*, *93*(4), 587-592.
Paternal Inheritance	Chloroplasts	Plants	*Pinus tabulaeformis*	Guo, F., Hu, S. Y., Yuan, Z., Zee, S. Y., & Han, Y. (2005). Paternal cytoplasmic transmission in Chinese pine (Pinus tabulaeformis). *Protoplasma*, *225*(1), 5-14.
Paternal Inheritance	Chloroplasts	Plants	*Pinus taeda* (loblolly pine)	Neale, D. B., & Sederoff, R. R. (1989). Paternal inheritance of chloroplast DNA and maternal inheritance of mitochondrial DNA in loblolly pine. *Theoretical and Applied Genetics*, *77*(2), 212-216.
Paternal Inheritance	Chloroplasts	Plants	*Pseudotsuga menziesii* (Douglas fir)	Neale, D. B., Wheeler, N. C., & Allard, R. W. (1986). Paternal inheritance of chloroplast DNA in Douglas-fir. *Canadian Journal of Forest Research*, *16*(5), 1152-1154.
Paternal Inheritance	Chloroplasts	Plants	*Sequoia sempervirens (coastal redwood)*	Neale, D. B., Marshall, K. A., & Sederoff, R. R. (1989). Chloroplast and mitochondrial DNA are paternally inherited in Sequoia sempervirens D. Don Endl. *Proceedings of the National Academy of Sciences*, *86*(23), 9347-9349.
Paternal Inheritance	Mitochondria	Plants	*Calocedrus decurrens* (incense cedar)	Neale, D. B., Marshall, K. A., & Harry, D. E. (1991). Inheritance of chloroplast and mitochondrial DNA in incense-cedar (Calocedrus decurrens). *Canadian Journal of Forest Research*, *21*(5), 717-720.
Paternal Inheritance	Mitochondria	Plants	*Cucumis melo* (melon)	Havey, M. J., McCreight, J. D., Rhodes, B., & Taurick, G. (1998). Differential transmission of the Cucumis organellar genomes. *Theoretical and Applied Genetics*, *97*(1), 122-128.
Paternal Inheritance	Mitochondria	Plants	*Cucumis sativus* (cucumber)	Havey, M. J. (1997). Predominant paternal transmission of the mitochondrial genome in cucumber. *Journal of Heredity*, *88*(3), 232-235.
Paternal Inheritance	Mitochondria	Plants	*Musa acuminata* (banana)	Fauré, S., Noyer, J. L., Carreel, F., Horry, J. P., Bakry, F., & Lanaud, C. (1994). Maternal inheritance of chloroplast genome and paternal inheritance of mitochondrial genome in bananas (Musa acuminata). *Current Genetics*, *25*(3), 265-269.

Paternal Inheritance	Mitochondria	Plants	*Sequoia sempervirens (coastal redwood)*	Neale, D. B., Marshall, K. A., & Sederoff, R. R. (1989). Chloroplast and mitochondrial DNA are paternally inherited in Sequoia sempervirens D. Don Endl. *Proceedings of the National Academy of Sciences*, *86*(23), 9347-9349.
Paternal Leakage	Chloroplasts	Plants	*Acacia decurrens*	Tilney-Basset, R. A. E. (1978). The inheritance and behaviour of plastids.
Paternal Leakage	Chloroplasts	Plants	*Acacia mearnsii*	Tilney-Basset, R. A. E. (1978). The inheritance and behaviour of plastids.
Paternal Leakage	Chloroplasts	Plants	*Antirrhinum majus*	Diers, L. (1971). Übertragung von Plastiden durch den Pollen bei Antirrhinum majus. *Molecular and General Genetics MGG*, *113*(2), 150-153.
Paternal Leakage	Chloroplasts	Plants	*Antirrhinum majus*	Reboud, X., & Zeyl, C. (1994). Organelle inheritance in plants. *Heredity*, *72*(2), 132-140.
Paternal Leakage	Chloroplasts	Plants	*Borago officinalis*	Sears, B. B. (1980). Elimination of plastids during spermatogenesis and fertilization in the plant kingdom. *Plasmid*, *4*(3), 233-255.
Paternal Leakage	Chloroplasts	Plants	*Brassicaceae*	Schneider, A., Stelljes, C., Adams, C., Kirchner, S., Burkhard, G., Jarzombski, S., ... & Koop, H. U. (2015). Low frequency paternal transmission of plastid genes in Brassicaceae. *Transgenic research*, *24*(2), 267-277.
Paternal Leakage	Chloroplasts	Plants	*Browallia speciosa*	Sears, B. B. (1980). Elimination of plastids during spermatogenesis and fertilization in the plant kingdom. *Plasmid*, *4*(3), 233-255.
Paternal Leakage	Chloroplasts	Plants	*Browallia speciosa*	Reboud, X., & Zeyl, C. (1994). Organelle inheritance in plants. *Heredity*, *72*(2), 132-140.
Paternal Leakage	Chloroplasts	Plants	*Chenopodium album*	Gasquez, J., Mouemar, A. A., & Darmency, H. (1985). Triazine herbicide resistance in Chenopodium album L.: occurrence and characteristics of an intermediate biotype. *Pesticide science*, *16*(4), 392-396.
Paternal Leakage	Chloroplasts	Plants	*Chlorophytum elatum*	Tilney-Basset, R. A. E. (1978). The inheritance and behaviour of plastids.
Paternal Leakage	Chloroplasts	Plants	*Chlorophytumc omosum*	Tilney-Basset, R. A. E. (1978). The inheritance and behaviour of plastids.
Paternal Leakage	Chloroplasts	Plants	*Cicer arietinum*	Corriveau, J. L., & Coleman, A. W. (1988). Rapid screening method to detect potential biparental inheritance of plastid DNA and results for over 200 angiosperm species. *American Journal of Botany*, *75*(10), 1443-1458.
Paternal Leakage	Chloroplasts	Plants	*Epilobium angustifolium*	Reboud, X., & Zeyl, C. (1994). Organelle inheritance in plants. *Heredity*, *72*(2), 132-140.
Paternal Leakage	Chloroplasts	Plants	*Fagopyrum esculentum*	Sears, B. B. (1980). Elimination of plastids during spermatogenesis and fertilization in the plant kingdom. *Plasmid*, *4*(3), 233-255.
Paternal Leakage	Chloroplasts	Plants	*Hypericum perforatum*	Corriveau, J. L., & Coleman, A. W. (1988). Rapid screening method to detect potential biparental inheritance of plastid DNA and results for over 200 angiosperm species. *American Journal of Botany*, *75*(10), 1443-1458.
Paternal Leakage	Chloroplasts	Plants	*Impatiens glandulifera*	Richter-Landmann, W. (1959). Der Befruchtungsvorgang bei Impatiens glandulifera royle unter Berücksichtigung der plasmatischen Organelle von Spermazelle, Eizelle und Zygote. *Planta*, *53*(3), 162-177.

Paternal Leakage	Chloroplasts	Plants	*Ipomoea nil*	Corriveau, J. L., & Coleman, A. W. (1988). Rapid screening method to detect potential biparental inheritance of plastid DNA and results for over 200 angiosperm species. *American Journal of Botany*, *75*(10), 1443-1458.
Paternal Leakage	Chloroplasts	Plants	*Nicotiana tabacum; N. plumbaginifoli* (tobacco cybrids)	Medgyesy, P., Páy, A., & Márton, L. (1986). Transmission of paternal chloroplasts in Nicotiana. *Molecular and General Genetics MGG*, *204*(2), 195-198.
Paternal Leakage	Chloroplasts	Plants	*Oryza sativa*	Dally, A. M., & Second, G. (1990). Chloroplast DNA diversity in wild and cultivated species of rice (Genus Oryza, section Oryza). Cladistic-mutation and genetic-distance analysis. *Theoretical and Applied Genetics*, *80*(2), 209-222.
Paternal Leakage	Chloroplasts	Plants	*Pisum sativum*	Corriveau, J. L., & Coleman, A. W. (1988). Rapid screening method to detect potential biparental inheritance of plastid DNA and results for over 200 angiosperm species. *American Journal of Botany*, *75*(10), 1443-1458.
Paternal Leakage	Chloroplasts	Plants	*Poa annua*	Darmency, H., & Gasquez, J. (1981). Inheritance of triazine resistance in Poa annua: consequences for population dynamics. *New Phytologist*, *89*(3), 487-493.
Paternal Leakage	Chloroplasts	Plants	*Solarium tuberosum (potato)*	Sears, B. B. (1980). Elimination of plastids during spermatogenesis and fertilization in the plant kingdom. *Plasmid*, *4*(3), 233-255.
Paternal Leakage	Mitochondria	Plants	*Brassica napus*	Erickson, L., & Kemble, R. (1993). The effect of genotype on pollen transmission of mitochondria in rapeseed (Brassica napus). *Sexual Plant Reproduction*, *6*(1), 33-39.
Paternal Leakage	Mitochondria	Plants	*Daucus carota*	Mandel, J. R., Ramsey, A. J., Holley, J. M., Scott, V. A., Mody, D., & Abbot, P. (2020). Disentangling complex inheritance patterns of plant organellar genomes: an example from carrot. *Journal of Heredity*, *111*(6), 531-538.
Paternal Leakage	Mitochondria	Plants	*Pinus banksiana* (jack pine)	Wagner, D. B., Dong, J., Carlson, M. R., & Yanchuk, A. D. (1991). Paternal leakage of mitochondrial DNA inPinus. *Theoretical and Applied Genetics*, *82*(4), 510-514.
Paternal Leakage	Mitochondria	Plants	*Pinus contorta* (lodgepole pine)	Wagner, D. B., Dong, J., Carlson, M. R., & Yanchuk, A. D. (1991). Paternal leakage of mitochondrial DNA inPinus. *Theoretical and Applied Genetics*, *82*(4), 510-514.
Paternal Leakage	Mitochondria	Plants	*Silene vulgaris*	McCauley, D. E., Bailey, M. F., Sherman, N. A., & Darnell, M. Z. (2005). Evidence for paternal transmission and heteroplasmy in the mitochondrial genome of Silene vulgaris, a gynodioecious plant. *Heredity*, *95*(1), 50-58.
Paternal Inheritance	Mitochondria	Viridiplantae	*Chlamydomonas reinhardtii*	Nakamura, S. (2010). Paternal inheritance of mitochondria in Chlamydomonas. *Journal of plant research*, *123*(2), 163-170.
Tertiary Parent	Mitochondria	Amoebozoa	*Dictyostelium discoideum*	Bloomfield, G., Paschke, P., Okamoto, M., Stevens, T. J., Urushihara, H. (2019) Triparental inheritance in Dictyostelium. *PNAS*, 116:2187–2192
